# Maternal internal migration and child growth and nutritional health in Peru: an analysis of the demographic and health surveys from 1991 to 2017

**DOI:** 10.1186/s12889-021-12452-7

**Published:** 2022-01-06

**Authors:** Emeline Rougeaux, J. Jaime Miranda, Mary Fewtrell, Jonathan C. K. Wells

**Affiliations:** 1 Childhood Nutrition Research Centre, UCL Great Ormond Street Institute of Child Health, London, UK; 2 CRONICAS Centre of Excellence in Chronic Diseases, Universidad Peruana Cayetano Heredia, Lima, Peru; 3 Department of Medicine, School of Medicine, Universidad Peruana Cayetano Heredia, Lima, Peru

**Keywords:** Migration, Stunting, Overweight, Double burden of malnutrition, Child health, Global health

## Abstract

**Background:**

Peru has historically experienced high rural-to-urban migration. Despite large reductions in undernutrition, overweight is increasing. Elsewhere, internal migration has been associated with differences in children’s growth and nutritional health. We investigated how child growth and nutritional status in Peru varied over time and in association with maternal internal migration.

**Methods:**

Using data from Demographic & Health Surveys from 1991 to 2017, we assessed trends in child growth (height-for-age [HAZ], weight-for-age [WAZ], weight-for-height [WHZ] z scores) and nutritional health (stunting, underweight, overweight) by maternal adult internal migration (urban [UNM] or rural non-migrant [RNM], or urban-urban [UUM], rural-urban [RUM], rural-rural [RRM], or urban-rural migrant [URM]). Using 2017 data, we ran regression analyses, adjusting for confounders, to investigate associations of maternal migration with child outcomes and the maternal and child double burden of malnutrition. We further stratified by timing of migration, child timing of birth and, for urban residents, type of area of residence. Results are given as adjusted predictive margins (mean z score or %) and associated regression *p*-values [p].

**Results:**

In 1991–2017, child growth improved, and undernutrition decreased, but large differences by maternal migration persisted. In 2017, within urban areas, being the child of a migrant woman was associated with lower WHZ (UUM = 0.6/RUM = 0.5 vs UNM = 0.7; *p* = 0.009 and *p* < 0.001 respectively) and overweight prevalence ((RUM 7% vs UNM = 11% [*p* = 0.002]). Results however varied both by child timing of birth (birth after migration meant greater overweight prevalence) and type of area of residence (better linear growth in children of migrants [vs non-migrants] in capital/large cities and towns but not small cities). In rural areas, compared to RNM, children of URM had higher HAZ (− 1.0 vs − 1.2; *p* < 0.001) and WAZ (− 0.3 vs − 0.4; *p* = 0.001) and lower stunting (14% vs 21%; [p < 0.001]). There were no differences by timing of birth in rural children, nor by time since migration across all children. The mother and child double burden of malnutrition was higher in rural than urban areas but no differences were found by maternal internal migration.

**Conclusions:**

Migration creates a unique profile of child nutritional health that is not explained by maternal ethnic and early life factors, but which varies depending on the pathway of migration, the child timing of birth in relation to migration and, for urban dwellers, the size of the place of destination. Interventions to improve child nutritional health should take into consideration maternal health and migration history.

**Supplementary Information:**

The online version contains supplementary material available at 10.1186/s12889-021-12452-7.

## Introduction

Although great progress has been made in tackling undernutrition at a global level, recent estimates indicate that among children under the age of 5 years, 22% are stunted and 7% wasted worldwide, and this now increasingly co-exists with rising levels of overnutrition [1]. Peru, like many other countries in Latin America, has historically been marked by persistent high rates of stunting (low height-for-age) but over the last decade it has succeeded in reducing the prevalence more than half (from 31% in 2000 to 13% in 2016 in children under 5 years) [2, 3]. However, the country has some of the world’s largest rural-urban disparities in nutritional outcomes and recent findings suggest that rural-urban and socio-economic inequalities in stunting have increased [3–6]. The prevalence of overweight in young Peruvian children has remained relatively stable since the turn of the century (at 10–12% in children under 5 years); although this varies greatly by region, and there has been a secular increase in overweight levels in adults [3, 7].

Early undernutrition may manifest itself in deficits in weight and/or linear growth. Stunting is the result of long term or chronic undernutrition (in utero or after birth) and/or infection hindering a child’s height potential, while wasting reflects a more immediate result of acute undernutrition or infection [8, 9]. Research is nonetheless increasingly suggesting that these two forms of undernutrition should not be viewed independently as they share many common risk factors, can co-occur, and repeated/chronic wasting could lead to stunting [10, 11]. Underweight is influenced by both weight and height and can reflect being wasted and/or stunted [8]. Inadequate nutrition during early periods of growth has been associated with changes in physiological and metabolic functioning and lower fat-free and fat mass and potentially fat mass distribution. This may in turn have consequences on physical health (e.g increased metabolism-related disease), mental health, cognition and social wellbeing over the lifecourse [8, 12, 13].

In contrast, overweight has been associated with diets and other behavioural and environmental factors that favour fat deposition, but it may also be associated with micronutrient deficiencies and hunger in certain contexts [9, 14]. Overweight in childhood and adolescence has been associated with increased risk of diabetes, cardiovascular disease and cancer [15], especially among those undernourished in earlier life [16]. Overweight may also co-exist with stunting at the individual and household levels [16]; both may also track into adulthood and affect offspring growth and wellbeing [8, 17]. In Peru however, the mother and child double burden of malnutrition, mostly characterised by overweight mothers with stunted children within the same households, has dropped from 19 to 12% between 1992 and 2012. With reported increases in maternal overweight, this decline is mostly due to changes in stunting during that period [18].

Different forms of malnutrition emerge within a broader sociocultural, environmental, economic, and political context which impacts food security and environment, eating practices and lifestyles, and access to health, sanitation, and hygiene services. Peru’s recent history of conflict in the 1980s and 1990s, which led to a great increase in rural-urban migration and urban informal settlements and exacerbated pre-existing high levels of poverty and food insecurity, shifted the context towards more malnutrition. Peru is also regularly affected by the El Niño–Southern Oscillation which severely disrupts regional weather patterns, causing natural disasters such as floods, food insecurity and disease, which may in turn contribute to deficits in child growth [19]. Future changes in weather due to global warming are predicted to increase the intensity and frequency of such events and population displacement and migration [20].

Studies from India, Mexico and Tanzania which explored the impact of migration on child growth in low- or middle-income countries, found that children born to internal (within-country) migrant mothers may have different nutritional outcomes than their non-migrant counterparts [21–26]. Notably, children of rural-urban migrant women tended to have a lower risk of undernutrition and a higher risk of overweight compared to the children of rural non-migrant women but the opposite when compared to children of urban non-migrant women [21–26]. Research into the impact of internal migration on children found that children who migrated with their parents (as opposed to children born after maternal migration) had lower survival [27]; however, it is unclear if this pattern may also be the case for other health outcomes. There have also been conflicting reports on whether children of more recent migrants are more vulnerable to different forms of malnutrition [22, 25].

Studies on adults in Peru show inequalities in anthropometric outcomes by internal migration status and suggest that moving to urban areas is associated with an increased risk of becoming overweight or obese [3, 28]. Findings of the Young Lives cohort of Peruvian children born in the early 2000s indicate that children of migrant women had lower levels of stunting and underweight than those of non-migrant mothers and that rural-urban migration might be associated with better child nutrition [29]. It is nonetheless uncertain how different pathways of internal migration are associated with growth and child nutritional health in this region and how this may vary by child migration status. Previous work from Peru suggests that geographic, socio-economic and prenatal factors are important determinants of child nutrition and may help explain how child nutrition varies by migration pathway [4–6, 30].

This study uses nationally representative Demographic and Health Surveys conducted in Peru between 1991 and 2017 and aims to quantify and assess:

- Aim 1: secular trends in child growth and nutritional status in Peru over time by maternal adult internal migration status (1991–2017).
- Aim 2: associations of maternal adult internal migration and child growth and nutritional status in the 2017 survey, considering in turn time since migration, child timing of birth and type of urban area of current residence.
- Aim 3: associations of maternal adult internal migration and the mother and child double burden of malnutrition (within children and between mothers and children living in the same households) in the 2017 survey.

The conceptual model hypothesizes that migration to urban areas will raise nutritional measures (reducing undernutrition but increasing overweight) while migration to a rural area will have the opposite effect; but these effects may be reduced after controlling for common pre-existing factors associated with both maternal adult migration and offspring growth and nutritional health and they may also differ by migration characteristics.

## Methods

### Data & study design

The data was obtained through the Peru Demographic and Health Surveys (DHS) or Encuesta Demográfica y Salud Familiar (ENDES) which were carried out by the Instituto Nacional de Estadística e Informática (INEI), Lima, Peru within the framework of the DHS Program [31].

The DHS surveys are cross-sectional nationally representative surveys focussing on women of child-bearing age (15–49 years) and their children under the age of 5 years. They consist of a questionnaire for the household and its members, exploring socio-demographic and economic characteristics, and a questionnaire for eligible women (established in the household questionnaire), exploring socio-economic characteristics, employment, domestic violence, health markers (maternal and child), family planning and pregnancies. Interviews also include anthropometric measurements of interviewed women and their children. Children under 5 years who are alive and residing in the household at the time of the interview and their mothers are the focus of this research; on average just under 20% of mothers had more than one child under 5 years in the survey and this ranged from 2 to 5 children. Mothers with children of multiple births were however excluded due to potential differences in early growth patterns for these children [32].

The Peru DHS from 1992 to 2017 were used in analyses of mean growth outcomes and the prevalence of forms of malnutrition and their trends over time. The 1986 survey was excluded as it did not contain the data relevant to this paper. The 1991–92, 1996 and 2000 surveys were standard DHS (usually conducted every 5 years), this then changed to a 5-year continuous DHS cycle with yearly surveys from 2003 to 2008 and then yearly continuous DHS surveys for all the following years. The DHS data from 2003, 2004 and 2006 (which formed part of the 2004–08 continuous DHS) did not contain any anthropometric measurements and therefore only data from 2005, 2007 and 2008 are included in the analyses of the 2004–2008 DHS cycle. The survey years, sample sizes and exclusions are detailed in Table 1.

**Table 1:** Maternal migration and child nutritional outcomes study sample (N), Peru DHS 1991–2017

DHS survey cycle	Total interviewed local resident women aged 15–49 years (A)	A + with singleton living resident children under 5 years (B)	A + B + complete child anthropometric data (C)	A + B + C + complete migration data (D)	A + B + C + D + repeat migrants excluded (E)	A + B + C + D + E + maternal childhood migration excluded (F)
1991–92	15,454	8430	7554	7554	7260	4606
1996	28, 251	15,813	14,379	14,357	13,923	9349
2000	27,002	12,577	11,263	11,243	10,929	7444
2004-08^a^	40, 572	15,953	10,075	10,049	9641	5770
2009	23, 692	9638	9099	9084	8757	5545
2010	22, 243	8659	8468	8432	8071	5134
2011	21, 963	8529	8426	8389	7951	5119
2012	23, 204	9012	8896	8862	8408	4788
2013	22, 381	8409	8292	8255	7925	4594
2014	24, 254	9034	8902	8878	8530	4887
2015	34, 994	22,751	22,495	22,411	21,571	12,549
2016	32, 495	20,240	19,977	19,885	19,075	10,924
2017	32, 541	20,499	20,272	20,180	19,394	10,913
Total	349,046	191,669	179,955	179,305	172,402	103,610

Footnotes: ^a^ Continuous survey with 5 yearly cycles; within this anthropometric data only collected in 2005, 2007 and 2008

The sample consisted of mothers who were usual residents in the city, town, or countryside where the interview took place, with singleton children under the age of 5 years living with them at the time of interview and who had complete anthropometric data (Table 1). Mothers who had not given any information on previous places of residence and who had lived abroad were excluded as their internal migration status could not be determined. Further exclusions were made on migration which are detailed further in Table 1 and in the measures section.

The DHS data for 1991–92 was collected using a two-stage sampling design for metropolitan areas (districts that form a city of more than 100,000 inhabitants) and a tri-stage sample design for non-metropolitan areas for each region of the country. In the tri-stage design, first districts were selected, then ‘conglomerates’ (geographic units consisting of households representing both urban and rural areas) within these and then households within the conglomerates. In the two-stage design, first conglomerates were selected and then households within these.

For DHS 1996 and 2000, a tri-stage design was carried out for the whole sample whereby first populated centres (such as cities, towns, villages etc) were systematically selected with probability proportional to population size sampling across all departments, then conglomerates selected within these and then households within conglomerates. From 2003 onwards, the DHS followed a two-stage design with first a selection of conglomerates in each department and then households selected within these. Further detail of sampling procedures can be found in the Final Reports for each survey (in Spanish) and in The Peru Continuous DHS Experience report (in English) available on the DHS website. All the analyses in this paper are weighted to account for this survey design using sampling weights provided by DHS for each survey cycle which allows for accurate population estimates. The weights also consider clustering in cases where mothers had several children under 5 years in the survey.

The DHS surveys were accessed from the DHS Program website for years 1991 to 2012 [31] and from the INEI website for subsequent years [33]. Procedures and questionnaires were reviewed and approved by the Inner City Fund Institutional Review Board (ICF IRB). The ICF IRB ensures that the survey complies with the U.S. Department of Health and Human Services regulations for the protection of human subjects (45 CFR 46), while INEI ensures it complies with laws and norms of Peru.

### Measures

#### Child growth and nutritional status

Children had their heights (using Shorr height boards) and weights (using SECA digital scales) measured by trained staff during the interview [34].

Height-for-age z scores (HAZ), weight-for-age z scores (WAZ) and weight-for-height z scores (WHZ) were created by the DHS team using WHO 2006 reference data for all years [9, 34]. We used these data to create cut-offs for stunting (defined as HAZ being lower than − 2 standard deviations [SDs] of the reference data median), underweight (WAZ lower than − 2 SDs of the reference data median), and overweight (WHZ higher than 2 SDs of the reference data median) for the DHS 2017 data descriptive analyses [9]. Wasting was not explored as previous research on the DHS indicated it to show very low prevalence in this population [3]. All other analyses used the z scores on a continuous scale.

#### Mother and child double burden of malnutrition

We defined a mother and child double burden of malnutrition at the household level following previous research on the topic and thus mothers and their children in the DHS were considered as having a double burden of malnutrition if any of the following applied [18]:

- the mother was overweight or obese with a child who was wasted, underweight or stunted
- the mother was thin with a child who was overweight
- the child was both stunted and overweight

Maternal weight categories were based on measures of height and weight taken during the survey [34] from which we calculated body mass index (BMI) as BMI = weight/height^2^ and then created cut-offs as thin (BMI < 18.5 kg/m^2^), healthy weight (BMI between 18.5 and 24.9 kg/m^2^), and overweight or obese (BMI ≥ 25 kg/m^2^).

#### Maternal adult internal migration

The DHS Peru included information on the nature of surveyed women’s childhood residence before 12 years of age and current residence (both categorized as capital or large city, small city, town or countryside, or abroad from DHS 2012 onwards) and length of time in the current area of residence. For the latter, women either responded either that they had ‘always’ lived there or gave a duration of current residence in years. Those who reported not having always lived in their current area of residence were also asked about their previous area of residence (using the same categories as childhood and current residence).

Using this information, we categorised women into different categories of internal migration. They were categorised as *non-migrants* if they said they had always lived in their current area of residence and there were no differences in the type of area of residence reported between their childhood and current residences. They were further classed either as urban non-migrant if they lived in an urban area (any city or town) or rural non-migrant if they lived in a rural area of residence (countryside). Women who did not say they had always lived in their current area of residence but who gave a length of time in current residence approximately the same as their current age and who showed no differences in type of area of residence over time were also considered non-migrant.

Women were classed as *migrants* if they stated they had not always lived in their current area of residence, or if they had said they always lived in their current area of residence but reported different areas for childhood and current residence. Migrant women were then categorised based on the type of area of childhood and/or previous residence as either urban-urban, rural-rural, urban-rural or rural-urban migrant. Repeat migrants (women who had moved between rural and urban areas more than once, i.e. rural-urban-rural or urban-rural-urban migrants) were excluded as the small size of this group made the statistical analyses impossible.

Although maternal migration at any age showed the same association with child nutritional outcomes as maternal migration in adulthood, the research in this paper focuses on migration in adulthood (18 years and over) as it has been suggested that environmental factors in the period prior to puberty may impact reproductive characteristics [35]. Moreover, focusing on adult migration allows for the adjustment of maternal factors in earlier life as potential confounders in the regression analyses.

Therefore, the results in this paper only explore *maternal adult internal migration* and pathways that include single urban-urban, rural-urban, rural-rural or urban-rural movements. Exclusions are shown in Table 1.

#### Covariates

Potential confounders were identified based on prior knowledge from research literature and plausible associations with the outcomes [4, 5, 36].

Variables were identified which were associated with both women’s migration in adulthood and child growth and nutritional outcomes and therefore may confound their association:

- Maternal self-identified ethnicity (interviewees were asked how they felt or considered themselves based on ancestry and according to their customs, responses were categorised as Quechua, other indigenous or native group [Aymara, Amazon, other], African descent [Black, Moreno, Zambo, Mulato, African People, or African descent], White, Mixed [Mestizo], or Other)
- Maternal level of schooling completed (classified as none, incomplete primary school, complete primary school, incomplete secondary school or complete secondary school)
- Maternal height (in centimetres)
- Child birth order (ranging from 1st to 15th)
- Maternal age (in years)

These measures also served as proxies for maternal childhood socio-economic circumstances as no direct information on this was available. Maternal age was also used as a proxy for exposure to particular conditions such as conflict and/or climatic events which may have influenced both likelihood of migrating and various aspects of maternal phenotype, which may in turn impact growth and nutritional health of offspring. Birth order reflected both maternal age at first birth and parity which may have influenced the decision to migrate as well as offspring physical health through maternal physical pathways.

Child age and sex were also added in the models as covariates and/or confounders depending on the analyses.

All these variables were provided in the DHS. They were based on maternal self-report, except for maternal height which was measured by trained staff at each survey round [34].

The relationship of maternal internal adult migration and child growth and nutritional status was also explored by the following measures:

- Time since maternal adult internal migration: using information on length of time in area of current residence provided by the DHS, a binary variable was created as long-term migrants (more than 5 years) and recent migrants (less than or equal to 5 years).
- Child timing of birth in relation to maternal adult internal migration: using information provided in the DHS on women’s age at birth of the DHS child, length of time in the current area of residence and women’s current age, further information could be determined on when the child was born in relation to their mother’s internal migration history. We classified children as being born before/during migration (if the mother’s age at birth of child was lower or equal to her age at migration; the range for this was up to 5 years before migration as only children under 5 years are including in the DHS) or after migration (if the mother’s age at birth of child was higher than her age at migration; this ranged from 1 to 30 years after migration).
- Current type of area of urban residence (Capital or large city, Small City, Town); provided by the DHS.

### Statistical analysis

#### Trends and absolute differences in child growth and nutritional status by maternal adult internal migration from 1991 to 2017 (aim 1)

First, trends in child growth and nutritional status in Peru were assessed over time stratified by maternal adult migration status. We calculated average HAZ, WAZ and WHZ and prevalence of stunting, overweight and underweight as well as their 95% confidence intervals (CI) for each maternal migration group at each DHS cycle from 1991 to 2017. Cycle-specific survey weights were applied at each DHS cycle as described further on. To assess statistical significance of trends over time regression analyses were used (linear for continuous outcomes and logistic for binary outcomes) in the survey-weighted pooled data with DHS survey cycle as the independent variable; the results are given as *p*-values for a difference between 1991 and 92 and 2017, for the full sample and each maternal migration group. Changes in the outcomes between 1991 and 92 and 2017 and associated p-values are also given in Additional File 1, Table A1.

#### Regression analyses of child growth and nutritional status using DHS 2017 (aim 2)

**Fig. 1:**
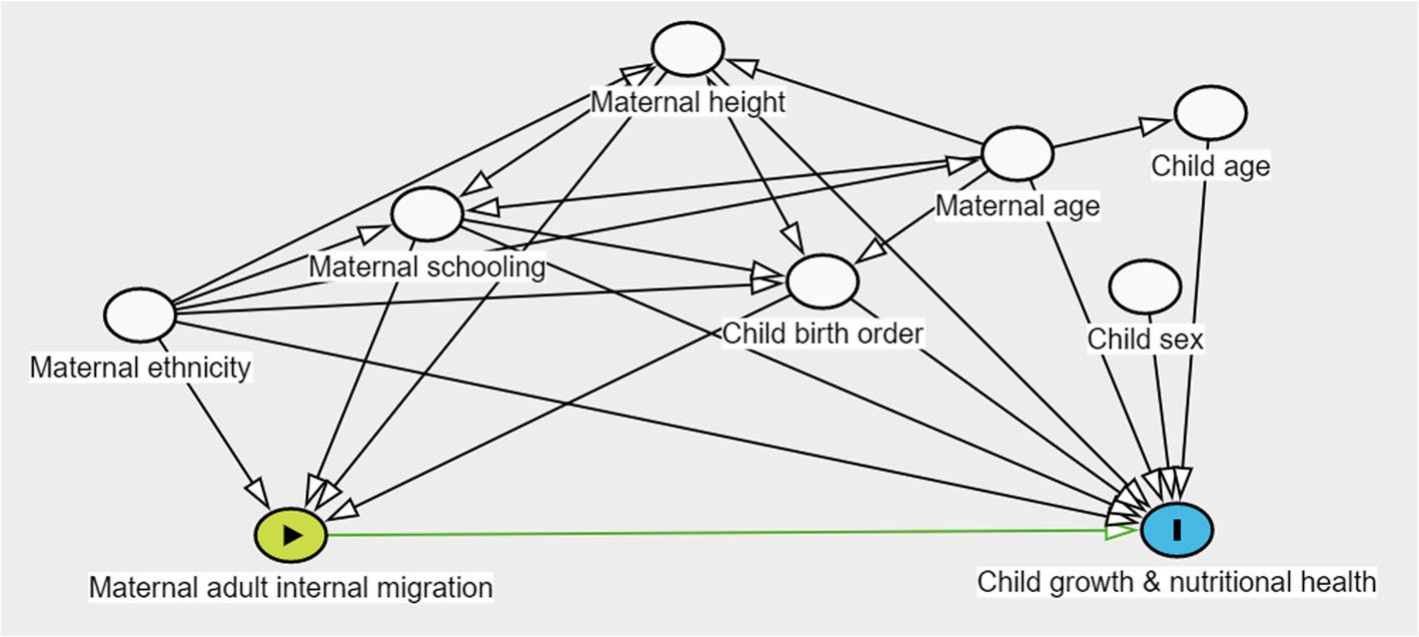
Direct Acyclic Graph for the association of maternal adult internal migration and child growth and nutritional health in Peru ^a^. Legend: ^a^ Created using DAGitty 3.0 (2020)

Using DHS data from 2017, survey-weighted regression was first carried out to explore the association between maternal adult internal migration status and child outcomes, adjusting for any potential confounders (Fig. 1) and using linear Ordinary Least of Squares (OLS) regression for growth outcomes and logistic regression for nutritional status outcomes. Urban non-migrants were the reference group; some of the analyses were however repeated with rural non-migrants as the reference group instead to explore differences within rural areas, this is specified in the results. Variables were identified as confounders using tests for association (Pearson’s Chi square tests for independence or ANOVA where appropriate).

These results were then explored stratified, in turn, by: time since maternal adult internal migration, child timing of birth in relation to maternal adult internal migration, and current type of urban area of residence (for current urban residents only).

As the DHS only includes children of ages ≤ 5 years and for groups to be comparable, the time since migration analyses were restricted to children born after migration and the child timing of birth analyses were restricted to children who were recent migrants (≤ 5 years).

Survey weighted descriptive statistics and results of the tests for association are given in Tables 2 and 3. Main results from the regression analyses are given as adjusted predictive margins in the text and figures, which were post-estimated after each adjusted regression analysis, and their associated regression *p*-values. These indicate the predicted outcome for a one unit change in the predictor variable, while keeping all other covariates as they are. For each growth outcome, this is the adjusted average z score, and, for each nutritional status outcome, this is the adjusted probability of occurrence, displayed as a percentage. Adjusted (and unadjusted) regression coefficients (for growth outcomes) and odds ratios (for nutritional status) and their 95% confidence intervals [CIs] are also reported in additional tables (Tables A2-A9, Additional File 1).

**Table 2:** Characteristics of Peruvian mothers and children under 5 years by maternal adult internal migration pathway in DHS Peru 2017; *N* = 10,910, weighted % (N) ^a, b^

		Urban non-migrant	Urban-urban migrant	Rural-urban migrant	Rural non-migrant	Rural-rural migrant	Urban-rural migrant	Total
Child age	< 6 months	8% (209)	6% (218)	8% (140)	8% (115)	8% (104)	9% (87)	7% (873)
	6-12mths	12% (267)	11% (384)	11% (196)	12% (180)	12% (145)	14% (123)	12% (1295)
	13-24mths	22% (548)	22% (712)	20% (331)	23% (297)	19% (213)	20% (211)	21% (2312)
	25-36mths	20% (499)	22% (662)	21% (332)	20% (283)	20% (231)	21% (190)	21% (2197)
	37-48mths	20% (501)	21% (664)	21% (362)	20% (274)	20% (229)	19% (193)	21% (2223)
	49-60mths	18% (433)	18% (603)	19% (348)	15% (223)	20% (239)	17% (164)	18% (2010)
Pearson’s Chi2 Test *p*-value		< 0.037						–
Child birth order	1st	41% (1005)	33% (1027)	25% (410)	30% (386)	15% (182)	33% (301)	33% (3311)
	2nd	32% (777)	34% (1061)	30% (502)	24% (336)	26% (289)	25% (242)	30% (3207)
	3rd	16% (397)	19% (655)	22% (375)	15% (211)	19% (231)	16% (171)	18% (2040)
	4th or more	10% (278)	14% (500)	23% (422)	31% (439)	39% (459)	27% (254)	19% (2352)
Pearson’s Chi2 Test p-value		< 0.001						–
Maternal age	15-17 yrs	1.4% (44)	0.0% (0)	0.0% (0)	2.6% (36)	0.0% (0)	0.0% (0)	1.4% (44)
	18-24 yrs	24.0% (613)	12.2% (446)	12.4% (206)	29.7% (394)	17.5% (206)	30.7% (261)	24.0% (613)
	25-29 yrs	22.5% (614)	25.9% (852)	23.9% (429)	20.8% (298)	25.5% (274)	26.2% (257)	22.5% (614)
	30-34 yrs	23.9% (558)	27.1% (857)	26.2% (426)	20.2% (265)	23.3% (277)	20.2% (213)	23.9% (558)
	35-39 yrs	18.5% (412)	22.0% (682)	21.7% (389)	15.2% (219)	19.2% (230)	14.1% (143)	18.5% (412)
	40-45 yrs	9.7% (216)	12.8% (406)	15.7% (259)	11.5% (160)	14.5% (174)	8.8% (94)	9.7% (216)
Pearson’s Chi2 Test *p*-value		< 0.001						–
Maternal schooling	None	0.1% (10)	0.4% (16)	2.3% (44)	5.1% (76)	7.8% (90)	1.3% (20)	1.8% (256)
	Incomplete primary	1.9% (72)	3.6% (137)	15.7% (249)	28.7% (381)	35.2% (407)	15.4% (149)	11.0% (1395)
	Complete primary	2.0% (92)	5.8% (180)	16.8% (287)	24.8% (316)	23.5% (262)	14.9% (130)	10.2% (1267)
	Incomplete secondary	12.7% (349)	13.9% (426)	16.6% (303)	19.9% (294)	14.2% (185)	19.3% (208)	14.9% (1765)
	Complete secondary	83.3% (1934)	76.3% (2484)	48.6% (826)	21.5% (305)	19.3% (217)	49.2% (461)	62.1% (6227)
Pearson’s Chi2 Test p-value		< 0.001						–
Ethnicity	Quechua	17% (467)	24% (909)	34% (403)	32% (565)	40% (562)	36% (707)	23% (3613)
	Other indigenous	1% (30)	4% (155)	6% (58)	11% (186)	7% (84)	6% (149)	3% (662)
	Black (Negro, Moreno, Zambo)	9% (251)	8% (241)	10% (77)	16% (168)	12% (105)	11% (137)	9% (979)
	White	6% (154)	7% (204)	7% (63)	9% (94)	7% (66)	9% (127)	7% (708)
	Mixed (Mestizo)	62% (1437)	51% (1610)	39% (320)	17% (213)	18% (208)	29% (469)	53% (4257)
	Other	2% (37)	1% (34)	0.2% (1)	1% (12)	2% (11)	2% (21)	2% (116)
	Don’t know	4% (134)	4% (169)	5% (71)	14% (143)	9% (145)	9% (126)	5% (788)
Pearson’s Chi2 Test *p*-value		< 0.001						–
Type of area of current residence	Capital/large city	79% (1290)	34% (383)	22% (133)	0% (0)	0% (0)	0% (0)	39% (1806)
	Small city	9% (486)	29% (1395)	27% (599)	0% (0)	0% (0)	0% (0)	14% (2480)
	Town	13% (681)	37% (1465)	51% (977)	0% (0)	0% (0)	0% (0)	20% (3123)
	Countryside	0% (0)	0% (0)	0% (0)	100% (1372)	100% (1161)	100% (968)	27% (3501)
Pearson’s Chi2 Test p-value		< 0.001						–
Geographic region of current residence	Metropolitan Lima	79% (1290)	34% (383)	22% (133)	0% (0)	0% (0)	0% (0)	39% (1806)
	Coast (excluding Lima)	14% (645)	30% (1190)	28% (507)	18% (198)	12% (108)	18% (127)	20% (2775)
	Highlands	4% (265)	20% (810)	36% (627)	59% (797)	60% (651)	43% (422)	26% (3572)
	Amazon Jungle	3% (257)	16% (860)	14% (442)	23% (377	23% (402)	39% (419)	15% (2757)
Pearson’s Chi2 Test p-value		< 0.001						–
Child timing of birth	Before/during maternal migration	–	23% (842)	20% (358)	–	24% (266)	35% (326)	24% (1792)
	After maternal migration	–	77% (2401)	80% (1351)	–	76% (895)	65% (642)	76% (5289)
Pearson’s Chi2 Test p-value		< 0.001						–
Time in current place of residence (migrants only)	5 years or less	–	50% (1719)	43% (751)	–	49% (569)	65% (614)	36% (2457)
	6 to 10 years	–	25% (790)	30% (505)	–	27% (316)	21% (207)	25% (3243)
	11 to 15 years	–	14% (422)	15% (260)	–	14% (161)	7% (79)	12% (1709)
	16 to 20 years	–	9% (242)	8% (138)	–	8% (91)	6% (56)	9% (1372)
	21 or more years	–	3% (70)	3% (55)	–	3% (24)	1% (12)	7% (1161)
Pearson’s Chi2 Test p-value		< 0.001						–
Total		36% (2457)	25% (3243)	12% (1709)	12% (1372)	9% (1161)	7% (968)	100% (10910)

Footnotes: ^a^ DHS survey-specific weights applied; ^b^ Sample covariate missing data (N): none

**Table 3:** Physical measurements of Peruvian mothers and children under 5 years by maternal internal adult migration pathway in DHS Peru 2017; N = 10,910; weighted % (N) or weighted mean (N) ^a, b^

		Urban non-migrant	Urban-urban migrant	Rural-urban migrant	Rural non-migrant	Rural-rural migrant	Urban-rural migrant	Total
**I. Child**								
Overweight (WHO cut-off)		12% (260)	9% (268)	6% (106)	3% (48)	4% (46)	4% (39)	8% (767)
Pearson’s Chi2 Test p-value		< 0.001						–
Underweight (WHO cut-off)		1% (52)	2% (63)	3% (46)	8% (115)	7% (67)	5% (43)	3% (386)
Pearson’s Chi2 Test p-value		< 0.001						–
Stunted (WHO cut-off)		6% (195)	7% (276)	11% (202)	32% (437)	30% (314)	17% (163)	13% (1587)
Pearson’s Chi2 Test p-value		< 0.001						–
**II. Mother**								
Mean height (in centimetres)		154 (2456)	153 (3241)	151 (1706)	151 (1372)	150 (1161)	151 (965)	152 (10901)
ANOVA test p-value		< 0.001						–
Weight (BMI cut-offs)	Healthy weight	30% (748)	30% (988)	31% (498)	46% (644)	39% (460)	44% (397)	34% (3735)
	Thin	1% (28)	1% (15)	< 1% (4)	< 1% (20)	1% (7)	1% (6)	1% (80)
	Overweight	38% (944)	43% (1384)	47% (812)	36% (485)	41% (480)	40% (393)	40% (4498)
	Obese	32% (736)	26% (854)	22% (391)	17% (223)	19% (214)	15% (169)	25% (2587)
Pearson’s Chi2 Test p-value		< 0.001						–

Footnotes: ^a^ DHS survey-specific weights applied; ^b^ Sample covariate missing data (N): maternal weight 10

#### Regression analyses of the mother and child double burden of malnutrition using DHS 2017 (aim 3)

Lastly, the mother and child double burden of malnutrition was explored across the different maternal adult internal migration groups. Logistic regression analyses were carried out, adjusting for confounders., and adjusted predictive margins were computed (as defined previously) giving the predicted percentage probability of the mother and child double burden of malnutrition; these are reported alongside associated regression p-values. Regression odds ratios (unadjusted and adjusted) and their 95% CIs are given in additional Table A10 (Additional File 1).

All analyses were carried out in Stata 15 (StataCorp LP, TX) using *svy* survey-specific commands which consider the complex sampling design of the DHS and include information on the primary sampling unit and household-level clustering, stratification in the survey sampling (e.g rural/urban within regions) and women’s sampling weights (which are based on household weights and response rates of individual women within each stratum). Predictive margins were estimated using the *margins* post-estimation command after each adjusted regression analysis.

In the given results, within each figure, the graphical representations are shown with a Y axis on the same scale to facilitate comparison. *P*-values < 0.05 were considered statistically significant.

## Results

### Secular trends in child growth and nutritional status by maternal adult internal migration: from 1991 to 2017 (aim 1)

#### Growth

Data from 1991 and 2017 indicate there was an overall increase in HAZ in children under the age of 5 years (with mean z scores of − 1.6 in 1991-92 and − 0.8 in 2017; *p* < 0.001). The increase in HAZ occurred, and was significant, in all migration groups but was greatest in children of rural-urban migrants and smallest in both children of rural-rural and urban-urban migrants (Additional File Table A1). However, mean HAZ remained negative compared to reference data for all years.

**Fig. 2:**
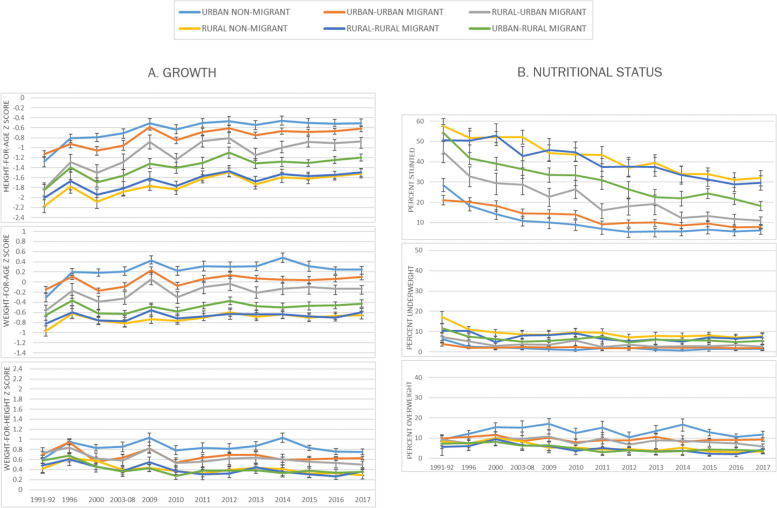
Growth and nutritional trends for children under 5 years by maternal adult internal migration in the DHS Peru 1991–2017 ^a^. Legend: I symbol indicates survey-specific mean/percentage 95% confidence intervals; * symbol indicates a p-value < 0.05 for regression comparisons of data for 1991–92 and 2017; ^a^ DHS survey-specific weights applied

From the early 1990s, differences in mean HAZ between maternal adult internal migration groups were observed which persisted over time (Fig. 2A). Children of mothers who were rural non-migrants and rural-rural migrants jointly had the lowest mean HAZ. Children of urban-rural mothers appeared to have better mean HAZ from 1996 onwards, compared to other rural children. Among urban dwelling families, children of rural-urban migrants and urban-urban migrants had lower mean HAZ than children of urban non-migrant mothers, but this difference was largest for the rural-urban group. Regardless, differences between urban and rural children persisted over time.

Overall mean WAZ increased slightly between 1991 and 2017 (with mean z scores of − 0.5 in 1991-92 and − 0.1 in 2017; *p* < 0.001). An increase occurred in all migration groups and was greatest in urban non-migrants and smallest in children of rural-rural migrants (Additional File 1 Table A1). Differences between different maternal migratory groups persisted over time with similar patterns and size differences to those seen for HAZ (Fig. 2A). However, WAZ was generally closer to the reference population than HAZ but below it for the most part.

Mean WHZ remained relatively stable overall over the DHS years and higher than the reference data for all children (with mean z scores of 0.6 on average in both 1991-92 and 2017; pooled data difference *p* = 0.869). However, there appeared to be a possible trend of widening differences between groups over time (Fig. 2A). More specifically, for children of urban non-migrants there was an increase in WHZ over time while there was a decrease for children in all other groups (albeit the change was not significant for urban-urban and rural-rural migrant groups, Additional File 1 Table A1). Generally, mean WHZ in children in rural settings was below that of urban children, but there were no differences within rural groups by maternal adult internal migration status, and differences both between rural and urban areas and within urban areas were very small compared to those seen in HAZ and WAZ.

#### Nutritional status

Between 1991 and 2017, there was a decrease in the prevalence of stunting by almost two thirds from 40 to 13% (*p* < 0.001). We see patterns of differences that reflect those seen in HAZ with the lowest level of stunting in urban children and the highest in rural children, with children of rural-urban and urban-rural migrant mothers having intermediate levels. Stunting declined significantly across all migration groups; the largest decline was seen in rural-urban migrants and the smallest in children of urban-urban migrants (Additional File Table A1). Over time, there was a decrease in differences within urban areas, but differences remained between urban and rural areas and within rural areas (Fig. 2B). There was an absolute difference in 2017 of 26% points between the most stunted group (rural non-migrant) and the least stunted (urban non-migrant) (Table 3).

During the same period, there was a decline in underweight from 9% in 1991 to 3% in 2017 (pooled data difference p < 0.001), mostly during the 1990s. The decline occurred across migration groups and was largest in rural non-migrant children (Additional File 1 Table A1). There were very small, or no apparent differences found by maternal adult internal migration status over time (Fig. 2B; see Table 3 for differences in prevalence in 2017).

Between 1991 and 2017, the prevalence of overweight in children under 5 years remained relatively stable overall at 8–9% (*p* = 0.737). Patterns by maternal adult internal migration status reflected those seen for WHZ. There was a small increase in overweight prevalence in urban non-migrant children, and a decline in all other groups, although this change was only significant in rural non-migrants (with a 5% point decline; p < 0.001). As a result, we see a widening of differences between groups over time (Fig. 2B). By 2017, we see a difference of 8% points between the rural groups and the urban non-migrant group, which had the highest prevalence of overweight in children under 5 years at 12% (Table 3).

### Associations of maternal and child internal migration and child growth and nutritional status in 2017 (aim 2)

#### Sample description

Characteristics of the sample from DHS 2017 and their association with maternal adult internal migration are given in Table 2.

Just over 50% of the mothers had been internal migrants in adulthood, mostly urban-urban migrants (25%) followed by rural-urban (12%), rural-rural (9%) and urban-rural migrants (7%). About a quarter of migrant mothers were relatively new migrants (in residence 5 years or less). Urban-rural migrants were the most likely to be newer migrants (≤ 5 years since arrival) and rural-urban migrants the least likely. Urban-rural migrant mothers were more likely to have had their DHS child before migration compared to other migrant groups (that were jointly similar in this respect).

Chi2 test *p*-values suggested that maternal adult internal migration was significantly associated with child age, maternal age, maternal schooling, birth order, current geographic region, current type of area of residence (urban residents only) and ethnicity (Table 2).

Note that all those who reported living in a ‘Capital or large city’ lived in the Lima Metropolitan Geographic Region.

Child nutritional status prevalence by maternal adult internal migration are given in Table 3. These reflect the patterns shown previously in Fig. 2 for DHS 2017. The largest absolute differences by maternal adult internal migration were found in stunting; differences in underweight and overweight were smaller but nonetheless significant. In contrast, the largest relative differences were found in underweight, followed by stunting and overweight. Across rural-dwelling groups in general (i.e rural non-migrants, rural-rural and urban-rural migrants), child overweight prevalence was lower, and child underweight and stunting prevalence were higher than for urban-dwelling groups; particularly when comparing non-migrant groups (Table 3). Within urban areas, children with a migrant mother, particularly a rural-urban migrant mother, were found to have a lower overweight but greater underweight and stunting prevalence compared to non-migrants. Differences in mean HAZ, WAZ and WHZ reflected patterns found for all three associated nutritional status outcomes as shown in Fig. 2.

Maternal migration was associated with maternal anthropometric outcomes (Table 3). The prevalence of overweight (excluding obesity) was higher for migrant mothers, particularly in urban areas and for rural-urban migrants (who had the highest prevalence overall at 47%). With obesity, rural-urban differences were larger, and the highest prevalence was found in urban non-migrant women at 32%. Mean maternal height was highest in urban non-migrants and urban-urban migrants; height was lowest in rural-rural migrants and similar in all other groups in rural non-migrants (Table 3). Shorter mothers appeared more likely to have shorter and stunted children, and overweight and obese mothers less likely to have stunted or underweight children but more likely to have overweight children (Chi2 tests suggest mother and child anthropometric measures were dependent with *p* < 0.001; data not shown here).

#### Findings

**Fig. 3:**
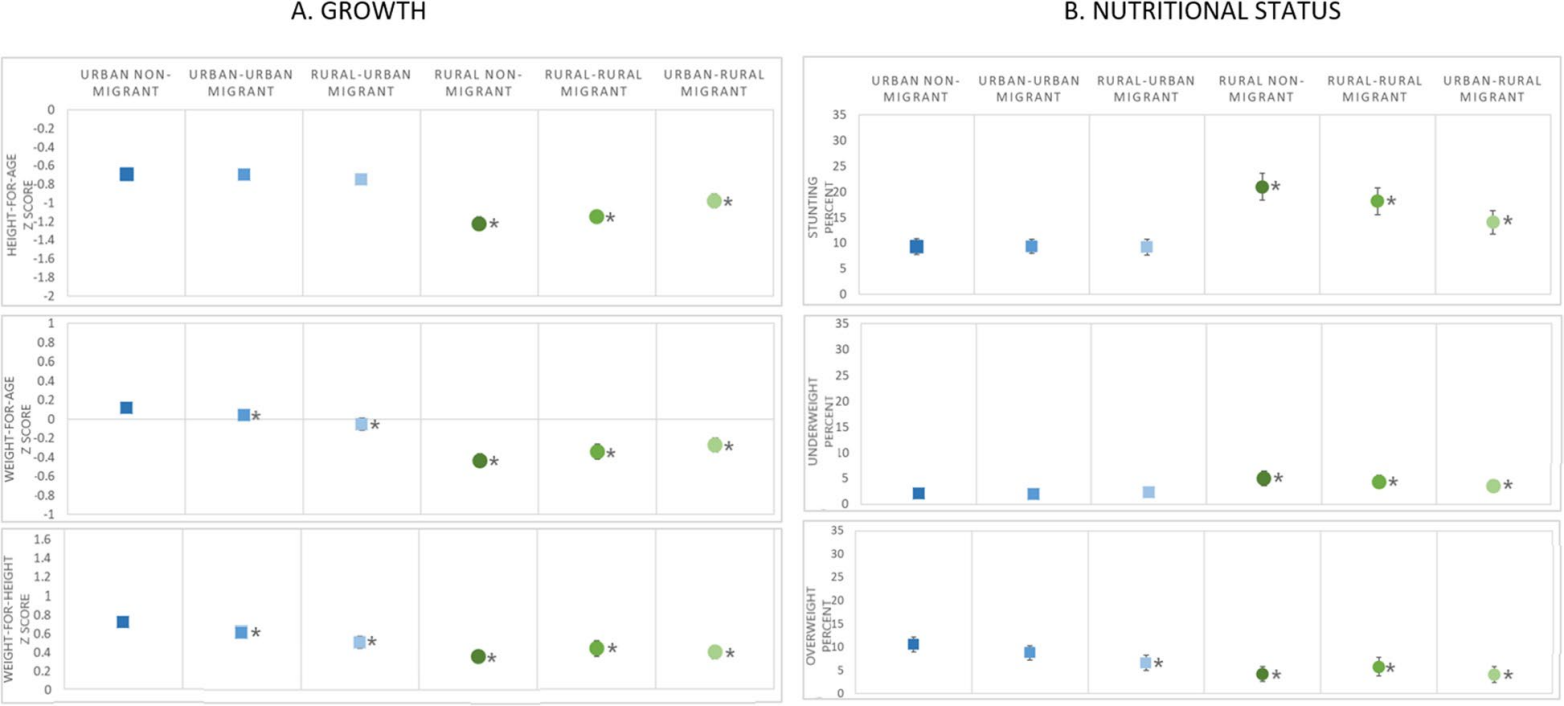
Growth and nutritional status for children under 5 years by maternal adult internal migration in the DHS Peru 2017; regression predictive margins ^a, b^. Legend: I symbol indicates 95% confidence intervals; ^a^ DHS survey-specific weights applied; ^b^ Adjusted for maternal ethnicity, schooling, height, age and child birth order, sex and age; * symbol indicates regression results significantly different from the reference group (urban non-migrants) with p-value < 0.05

Figure 3 presents predictive margins from the regression of maternal adult internal migration and child growth and nutritional status outcomes in the DHS 2017, after adjusting for confounders (maternal ethnicity, height, schooling, age and child birth order). Within the figure, panel A shows children’s predicted mean HAZ, WAZ and WHZ and panel B shows children’s predicted probability of stunting, underweight and overweight as a percentage. The asterisks indicate regression *p*-values < 0.05 for comparisons with the reference group (urban non-migrant). Regression coefficients (for growth outcomes) and odds ratios (for nutritional status) and their 95% CIs are also given in the additional Tables A2 and A3 (Additional File 1).

After adjusting for confounders, significant differences remained compared to the reference group (urban non-migrant) for all outcomes (Fig. 3). The results indicated overall that children living in rural areas (shown as green circles, Fig. 3) were more likely to have lower mean HAZ, WAZ and WHZ and overweight prevalence but a greater probability of stunting compared to children in urban areas (shown as blue squares, Fig. 3). Within urban areas, we saw significant differences between migrants and non-migrants; notably children of rural-urban mothers had lower mean WAZ and WHZ (both by 0.2 z scores, p < 0.001) and probability of overweight (by 4% points; *p* = 0.002) compared to urban non-migrants. All rural children had significantly lower mean HAZ, WAZ, WHZ and probability of overweight, and higher probabilities of stunting and underweight when compared to urban non-migrants (Fig. 3). Analyses were repeated with rural non-migrants as the reference group in order to assess differences within rural areas. This showed that children of urban-rural migrants had higher mean HAZ (0.2 z scores, p < 0.001) and WAZ (0.2 z scores, *p* = 0.001) and lower probability of stunting (by 7% points; p < 0.001) than rural non-migrants.

**Fig. 4:**
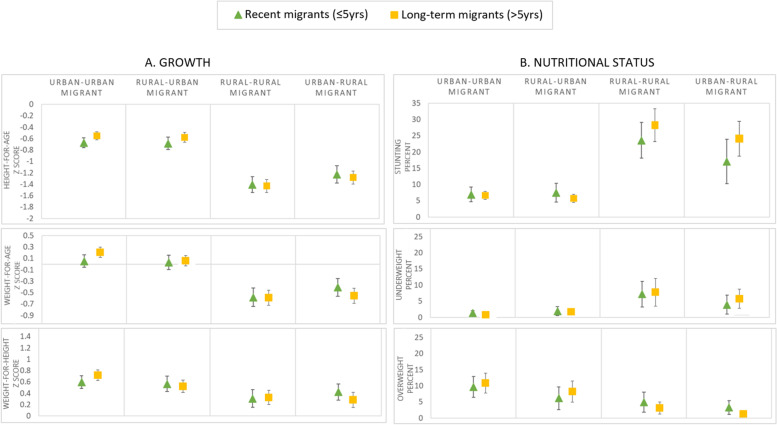
Growth and nutritional status for children under 5 years by maternal adult internal migration and timing of migration in the DHS Peru 2017; regression predicted margins, restricted to children born after migration *N* = 5284 ^a, b^ . Legend: ^a^ DHS survey-specific weights applied; ^b^ Adjusted for maternal ethnicity, schooling, height, age and child birth order, sex and age; I symbol indicates 95% confidence intervals; * symbol indicates regression results significantly different from the reference group (long-term migrants) with p-value < 0.05

Analysing the migrant women only, Fig. 4 shows comparisons of child growth and nutritional status by time since migration (recent migrants who have been in their current residence 5 or less years versus long-term migrants who have been in their current residence over 5 years). This was restricted to children born after migration as children born before maternal internal migration were all new migrants due to the sampled ages of DHS children. After adjusting for confounders (maternal ethnicity, schooling, height and age and child birth order), we no longer see any significant differences (Fig. 4; Additional File 1 Tables A4 and A5).

**Fig. 5:**
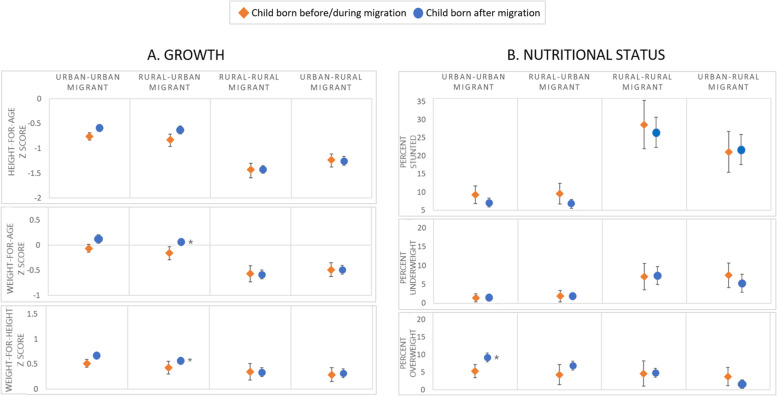
Growth and nutritional status for children under 5 years by maternal adult internal migration and child timing of birth in the DHS Peru 2017; regression predicted margins, restricted to recent migrants only [≤5 years since arrival] N = 3647 ^a, b^ . Legend: ^a^ DHS survey-specific weights applied; ^b^ Adjusted for maternal ethnicity, schooling, height, age and child birth order, sex and age; I symbol indicates 95% confidence intervals; * symbol indicates regression results significantly different from the reference group (born before/during maternal migration) with p-value< 0.05

Still analysing the migrant women only, Fig. 5 presents adjusted predictive margins for children stratified by their timing of birth and restricted to children of recent migration only. The asterisks indicate regression *p*-values < 0.05 for comparisons with the reference group (children born before/during migration). Unadjusted and adjusted regression coefficients and odds ratios are also given in additional Tables A6 and A7 (Additional File 1).

In rural-urban mothers, the adjusted results show a greater mean WAZ and WHZ for children born after migration compared to before/during migration (by 0.2 z scores; [*p* = 0.02] for both). We also see evidence of a higher probability of being overweight in children born after migration to urban-urban, and possibly rural-urban mothers, compared to those born before/during migration (by 4% points in both groups; p = 0.02 and 0.05 respectively)). There were no significant differences for these outcomes in other migration groups or for linear growth and stunting in any of the groups (Fig. 5).

**Fig. 6:**
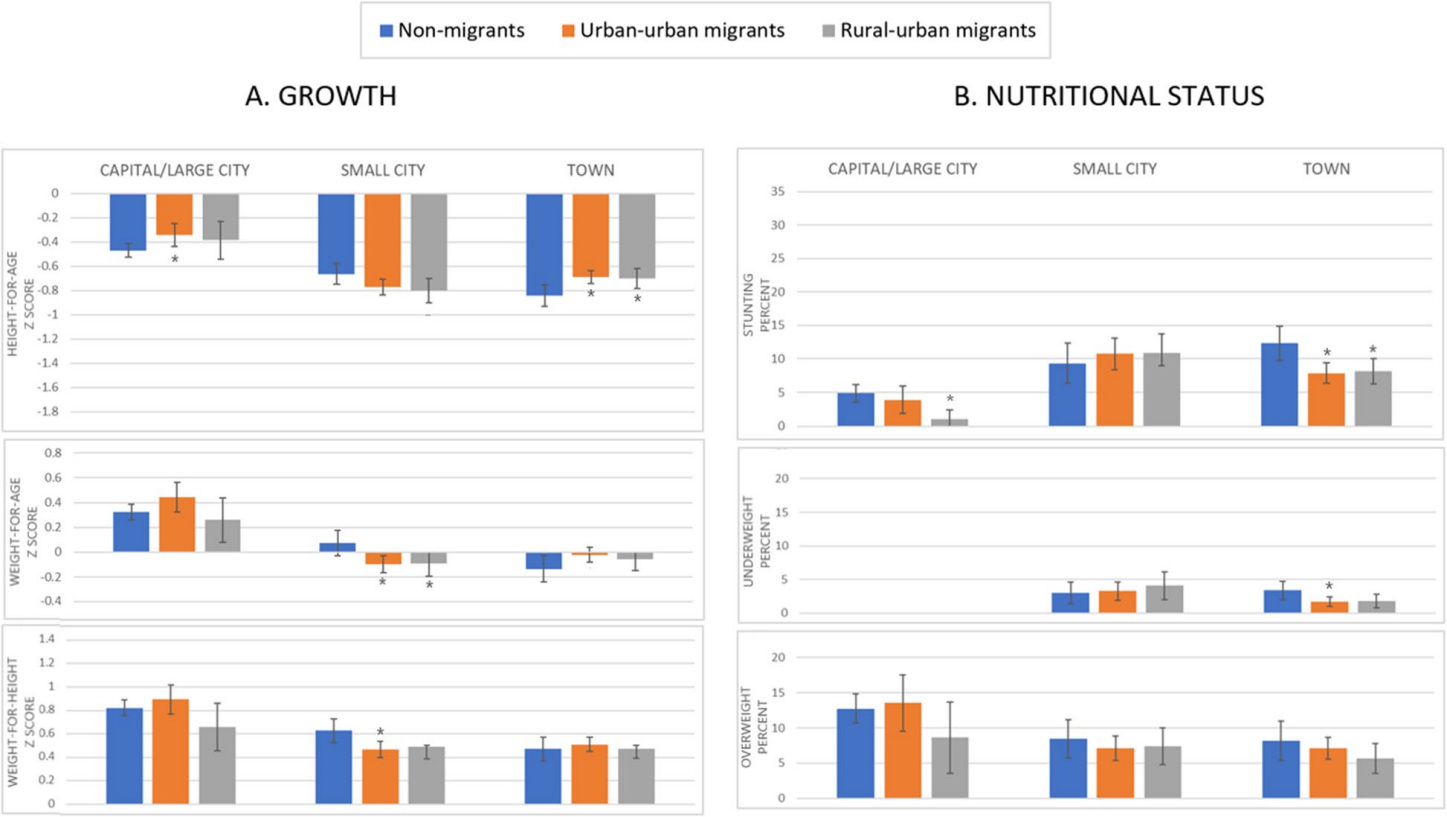
Growth and nutritional status for children under 5 years by maternal adult internal migration and current type of area of residence in urban residents in the DHS Peru 2017; regression predicted margins, *N* = 3647 ^a, b^. Legend: ^a^ DHS survey-specific weights applied; ^b^ Adjusted for maternal ethnicity, schooling, height, age and child birth order, sex and age; I symbol indicates 95% confidence intervals; * symbol indicates regression results significantly different from the reference group (urban non-migrants) with p-value< 0.05

Overall, child outcomes by current type of area of urban residence suggested that living in Capital/large cities was associated with better linear growth and lower levels of stunting, but also greater weight growth and higher levels of overweight, compared to living in small cities and towns. Exploring these outcomes by maternal adult internal migration showed variations by type of area of residence (Fig. 6). Figure 6 reports adjusted predictive margins as before, with the asterisks indicating regression p-values < 0.05 for comparisons with the reference group (urban non-migrant). While in Capital/large cities and towns, there was some evidence that children of migrants were likely to have better linear growth (significant in the urban-urban migrant group in Capital/large cities [*p* = 0.023] and all migrants in towns [UUM *p* = 0.004, RUM *p* = 0.021]) and lower stunting (significant in the rural-urban migrant group in Capital/large cities [*p* = 0.031] and all migrants in towns [UUM p = 0.001, RUM *p* = 0.012]), in small cities we do not find significant differences for these outcomes (only HAZ was borderline at *p* = 0.05 for both UUM and RUM). There was insufficient power to assess patterns in underweight in Capital/large cities. However, children of migrants in small cities had a lower WAZ [UUM *p* = 0.006, RUM p = 0.031] and WHZ [UUM only, p = 0.012] compared to non-migrants but there were no differences for these outcomes in other types of urban areas. The unadjusted and adjusted regression coefficients and odds ratios are given in additional Tables A8 and A9 (Additional File 1).

### Maternal internal migration and the mother and child double burden of malnutrition in 2017 (aim 3)

Across the Peru DHS 2017 sample (described previously in aim 2 results), we found a burden of 7.4 % (*n* = 865) double malnutrition for mothers and children under 5 years in Peru in 2017. Most of this took the form of overweight/obese mothers with underweight/wasted/stunted children. Only one mother in the sample was found to be thin with an overweight child and only 0.2% of children were both simultaneously categorised as overweight and stunted (*n* = 29).

**Fig. 7:**
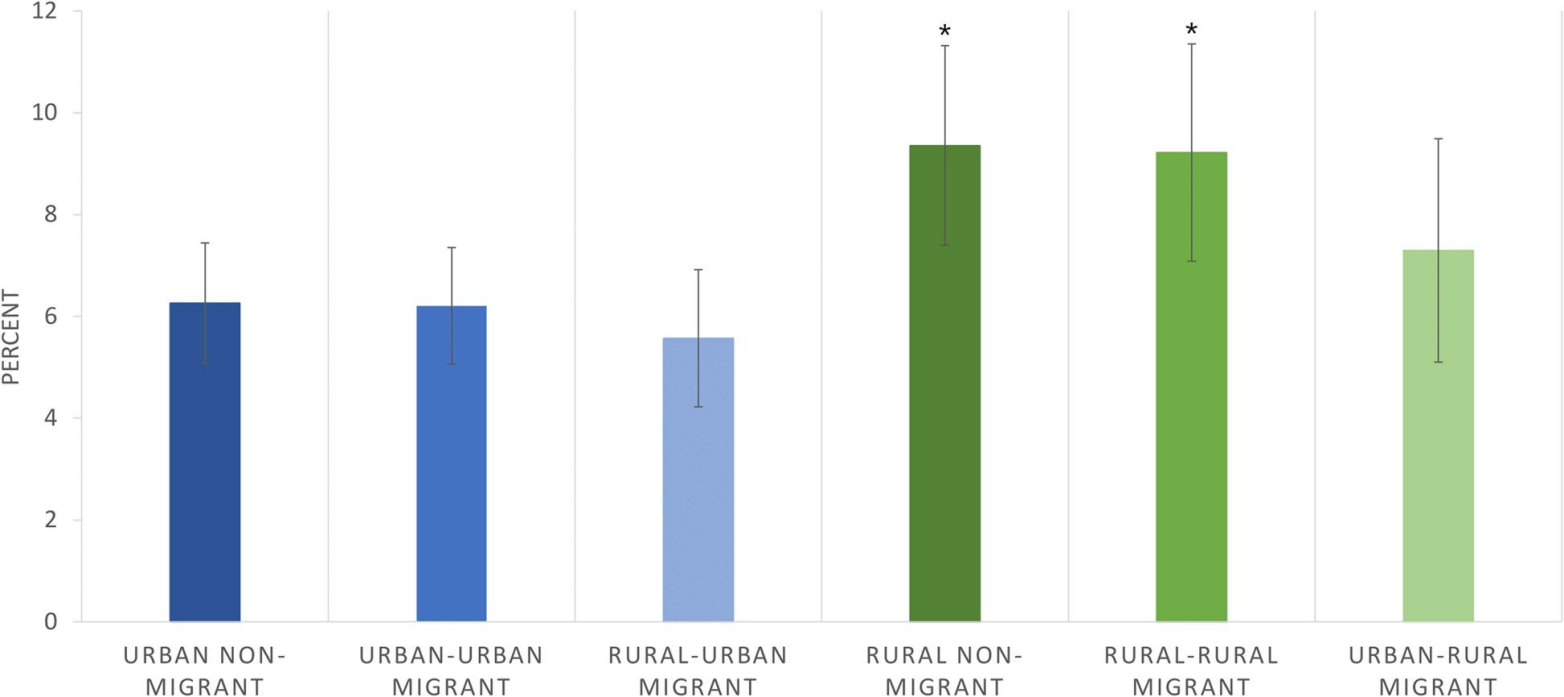
The double burden of malnutrition in mothers and children under 5 years by maternal adult internal migration status in the DHS Peru 2017; regression predicted margins ^a, b^. Legend: ^a^ DHS survey-specific weights applied; ^b^ Adjusted for maternal ethnicity, schooling, height, age and child birth order, sex and age; I symbol indicates 95% confidence intervals; * symbol indicates regression results significantly different from the reference group (urban non-migrants) with p-value< 0.05

After adjusting for confounders (maternal height, schooling, age and ethnicity and child birth order) both the predicted prevalence and differences were reduced. Figure 7 shows adjusted predictive margins; the asterisks indicating regression p-values < 0.05 for comparisons with the reference group (urban non-migrant). There remained significantly higher likelihood of mother and child double malnutrition in rural non-migrants and rural-rural migrants (with a 3% point higher probability; *p* < 0.05 in both groups) compared to the reference group (urban non-migrants), however there was no longer any evidence of differences within either urban or rural areas (when rural non-migrants were used as the reference group). Unadjusted and adjusted odds ratios can be found in Table A10, Additional File 1.

## Discussion

This paper sought to explore how maternal adult internal migration was associated with child growth and nutritional status in Peru. Addressing our first aim, we found secular changes in both child growth and nutritional status over time in Peru, however these changes for the most part occurred regardless of maternal adult internal migration status. Broadly across time, children of migrant women in urban areas had poorer linear growth and greater levels of stunting but lower levels of overweight compared to urban non-migrants, with the opposite pattern in rural areas. Addressing our second aim, more in-depth analyses of recent data from 2017 showed that these differences were partly explained by confounding factors associated with maternal migration and child growth and nutritional status. Differences remained, however, suggesting that women’s internal migration may influence offspring anthropometry. Addressing our third aim, we found no differences in the risk of mother and child double burden of malnutrition between migrant and non-migrant women neither within rural nor urban areas. We discuss these findings in greater detail below and then consider both future implications and what mechanisms may underlie the patterns found.

### Changes over time

Between 1991 and 2017 there was a steady increase in HAZ, a smaller increase in WAZ (mostly in the 1990s) and a smaller decrease in WHZ in Peruvian children under 5 years. This was accompanied by a large decline in stunting across time and a small decline in underweight (in the 1990s), with little change in the prevalence of overweight. These secular trends reflect the findings shown in previous DHS analyses [3, 6]. Reductions in stunting have been attributed to multi-sectorial poverty-reduction efforts implemented during that period [2, 6].

WHZ was overall higher than reference data for all children across the DHS and, although WHZ and overweight did not increase over time in DHS children under 5 years, we have seen increasing levels of overweight and obesity at older ages in Peru and across the Latin America region [28, 37, 38]. Furthermore, there appeared to be a mean shift towards positive WAZ for urban children over time. The positive WHZ trend may reflect historically low WHZ which could have slowed linear growth, subsequently allowing for recovery of WHZ using energy saved by slowing linear growth [10]. However, as Peru continues through its nutrition transition and food environments and behaviours change in the region [6, 37, 38], the prevalence of overweight will likely increase as the children increase in age. Indeed, previous findings in the Young Lives Peru cohort study of children indicate that this will likely happen both as the individual children grow older and across birth cohorts over time, particularly for those in living in urban areas [37].

While there were improvements overall, differences persisted between maternal adult internal migration groups over time for linear and weight growth and stunting, whereby urban areas showed better growth and lower prevalence of undernutrition, but higher prevalence of overweight compared to rural areas with migrants falling between urban and rural non-migrant groups. Children of urban-urban migrant mothers were the most like urban non-migrants while children of rural-rural migrants were the most like rural non-migrants. Children of rural-urban and urban-rural migrant women had outcomes between these two groups (urban-urban and rural-rural) but nonetheless displayed differences; the rural-urban group being more similar to the rural-rural group and the urban-rural group to the urban-urban group.

For WHZ and overweight, there was some evidence of a widening of differences by maternal migration groups over time driven by increases in urban non-migrants and decreases or no change in all other groups. This contrasts with findings in Peruvian adults which showed that obesity had increased across most migration groups and particularly in rural areas [6, 28]. It is therefore possible patterns would have been different in older children.

### Associations of maternal internal migration and child anthropometry in 2017

Further analyses carried out on the DHS for 2017 (addressing aim 2) showed that there were significant differences between mothers in different adult internal migration groups in terms of markers of early maternal socio-economic and nutritional environment (reflected in maternal schooling, age, ethnicity, height and child birth order). When these were considered, the predicted differences in child growth and nutritional health by maternal migration status were reduced but patterns were maintained for the most part. This supports findings that maternal phenotype and capital are important determinants of offspring growth and health [39–41] and justified our more detailed analyses that adjusted for such factors where data were available.

Overall, after adjusting for confounders, children in rural areas remained more likely to be worse off than children in urban areas in terms of linear growth and undernutrition (mostly stunting) but better off in terms of lower overweight prevalence. Among rural-dwelling children, having a migrant mother, particularly an urban-rural one, was associated with greater linear growth and lower likelihood of undernutrition (mostly stunting) with no differences for overweight or by timing of birth (reflecting rural or urban birth).

In contrast, in urban areas, having a migrant mother, particularly a rural-urban one, was associated with lower weight growth and overweight prevalence compared to non-migrants but there were no differences for linear growth and stunting. This was particularly the case if a child was born rurally and migrated subsequently to an urban setting; whereas children of urban-urban mothers and children of rural-urban mothers who were born after migration showed similar mean WHZ and overweight prevalence.

There was little evidence of any difference between children of recent and of long-term migrant mothers after taking confounders into account. Previous research in India had found that greater duration since maternal rural-urban migration was associated with higher levels of child undernutrition [22]; others have however argued that it very much depends on the context and that longer urban exposure is likely to increase obesogenic exposures [25]. Indeed, in adults in Peru, a longer time in urban residence was associated with a higher probability of being obese [28]. The small sample sizes in our analyses and young ages of the children may explain why we found no associations.

Longitudinal research is needed to explore whether as children of internal migrant women age, they become more like their urban non-migrant peers or instead retain anthropometric differences associated with maternal migration from rural areas. The data on mothers suggests that an earlier rural exposure is not necessarily protective against becoming overweight later, indeed migrant mothers in 2017 had higher levels of overweight than non-migrants within both urban and rural areas, with the highest across all groups found in rural-urban migrant mothers. However, the obesity prevalence was higher in the urban non-migrants (at 32%, compared to 26 and 22% in urban-urban and rural-urban migrant mothers respectively). Research on adults in the Peru Migrant Study (up to 2012–13) shows there may be variations by type of obesity with rural-urban migrants having the highest risk of central obesity [42].

For those living in urban areas, differences between migrants and non-migrants may differ by the type of area of residence; for example, children of migrants in capital/large cities and towns had better linear growth than non-migrants but not in small cities where patterns were in the opposite direction but not significant. The lack of significance here and for other outcomes may be due to the small sample sizes. It is therefore important to consider level of urbanization in child health and nutrition research and policy, not only because of variations in outcomes but also because the needs of migrant and non-migrant may vary.

### The mother & child double burden of malnutrition

The double burden of malnutrition analyses (aim 3) showed that mothers and children in rural areas had the highest risk of the double burden of malnutrition, mostly characterised as an overweight or obese mother with a stunted child, though there were no differences by maternal migration status after adjusting for confounders. Another study conducted in the Brazil DHS from 2006 found no differences in the mother and child double burden of malnutrition between rural and urban areas [43]; it is nonetheless possible that differences have emerged since.

Indeed, research by Popkin et al. shows that, with changes in global food systems and health behaviours, the double burden of malnutrition in LMICs is increasingly being concentrated in rural areas [16]. This highlights the need for social programmes in rural areas to consider both under- and over-nutrition at the household level, as it has previously been suggested that such programmes in Latin America remain primarily focussed on tackling undernutrition in children [36]. A study exploring the mother and child double burden of malnutrition in the DHS for Peru over time showed that while there had been an overall decline in prevalence, there had been an increase in undernourished children with overweight or obese mothers [44].

### Potential mechanisms

Together, the results of this paper suggest that maternal migration may impact offspring growth and nutritional health in different ways; this may be due to both exposure to rural or urban environments and the process of migrating itself. While migrating to cities may lead to new or better opportunities for employment and education and higher standards of living, it may also come with a loss of social support, barriers to accessing services, and stressful and hazardous work and living conditions [45–50]. Differences in these factors could explain variations in child health and survival by migration status [26, 51, 52].

It has recently been suggested that pollution may play a role in stunting [53] and previous research in Peru found that migrants in Greater Lima had a 10-fold increase in exposure to larger particulate matter (PM2.5) compared to non-migrants [54]. We did not find higher levels of stunting in children of migrants compared to non-migrants in larger cities but further investigation should compare child growth in areas with different levels of pollution within cities and at different ages. Changes in altitude with migration could potentially also play an important role in Peruvian child growth [55] and should also be further explored. Social networks during migration are likely important as well, for example in Indonesia it was found that rural-urban migration only negatively impacted adult mental health if it occurred alone rather than with family [47]. We did not however have information on these factors in the DHS.

Research suggests that stunting is mainly established by 24 months and therefore that interventions should ideally intervene with this critical window [39]. However more recently it was suggested that there may be opportunities beyond that with some interventions in LMICs showing improvements in height at later ages [39]. As they become older, it is possible that migrant and non-migrant children catch up with each other in stature, although differences will likely remain [56]. Research comparing highland and lowland (migrant) Peruvian children found differences in body lengths that were maintained throughout the ages studied up to 14 years [55]. Furthermore, early stunting, catch-up growth and rapid weight gain (which can follow early undernutrition) could predispose to greater fat mass later on [57, 58]. Low stature in children may reflect a low homeostatic metabolic capacity which could predispose children to ill health in the future, particularly when exposed to urban obesogenic environments (high metabolic load) [59]. Based on higher levels of overweight at older ages in Peru and research on women in the DHS showing increasing level of obesity with time since migration, the burden of obesity in Peruvian children will likely increase as they age and, in the case of migrants, acculturate to their new environment [28, 42, 60, 61].

While moving from rural to urban areas may improve access to food, it may also lead to food insecurity for some, as well as overeating, poorer quality food, changing food habits and/or sedentary behaviours, which may lead to obesity and related disease [26, 60, 62–67]. In Peruvian adults it was found that rural-urban migration was associated with an increased risk of becoming obese, developing inflammation, and metabolic disease, but not hypertension, type 2 diabetes and higher HbA1c or mental health disorders [49, 60, 68–70], perhaps reflecting benefits of an early rural exposure, as we discuss further on. The risk was increased with greater time in urban place of destination and possibly greater age at migration, although results were conflicting for the latter [60, 68, 69].

The negative impacts of urban migration are likely to be greater in slums or informal settlements where barriers to healthier foods may be higher [45, 71–73]. Findings from the Young Lives cohort of Peruvian children has shown that greater stunting is associated with poor access to sanitary facilities and greater food insecurity [61]. Qualitative research carried out in Peru’s ‘young towns’, peri-urban towns created informally by migrants, indicated that lack of green spaces and community resources, safety and the unhealthy food environment created barriers to good nutritional health [74].

An early exposure to particular gut microbes (‘Old Friends’) and various organism in the rural environment may influence immune system regulation and inflammatory processes and lead to rural and urban differences in child health [75]. Research has suggested that gut health may be important for linear growth in early life [39]. Rural to urban migration may possibly influence nutritional health through these pathways as well. Rook et al. found that an early rural (low-income country) exposure confers some benefits to immune regulation and various aspects of physiological health, thus reducing the risk of developing chronic inflammatory and psychiatric conditions after migration to a high-income urban environment [76, 77]. However, in contrast to the findings on metabolic health in other research, later migration was associated with greater benefits [76]. These effects of an early rural exposure may possibly explain why children of rural-urban migrants appeared to have lower levels of overweight. Internal rural to urban migration may impact other aspects of child health such as body composition, epigenetic ageing, inflammation, and hormonal profiles which are not available in existing data and would warrant further investigation.

### Strength and limitations

The DHS framework provides data that is collected using piloted and well-designed surveys following best practice for study design and data collection. As the surveys are carried out consistently through DHS/ENDES, this offers a large and nationally representative dataset that is comparable over time. The surveys also offer a wealth of information on both children and their mothers. This allowed us to explore migration and child nutritional outcomes as well as several maternal, socio-economic and environmental covariates. Three child nutritional outcomes are available, and the z scores are calculated by DHS using WHO 2006 reference data for *all* DHS surveys allowing for comparison across years.

The anthropometric data for these outcomes are collected by trained professionals using widely used and appropriate equipment. Interviewers verified some of the current information such as current residence making the data unlikely to be biased. Response rates were high for all surveys and the level of missing data was low for the measures used in this research.

The use of self-report for past area of residence and time in current area of residence could nonetheless have introduced some bias into the results (such as recall bias, particularly with increased time since migration). Different perceptions of what should be classified as a town or city when recalling previous area of residence could also have introduced bias. However, the relationship between maternal adult internal migration and child nutritional outcomes was relatively similar over time in different groups of women. Analyses were repeated using a measure of lifetime internal migration, and the patterns of association were like those using adult internal migration. Interviewers verified current residence making these data unlikely to be wrong. As sensitivity analyses, the statistical analyses carried out in DHS 2017 were repeated for the previous DHS survey and the results were similar.

The categories of migration used in the paper bring together a number of possible pathways of migration (when considering capital, city, town and countryside and previous and current areas of residence) which could not be explored in the regression models due to lack of power and which were therefore simplified into categories based on urban and rural residence instead, similarly to other research. However further exploration of the data (not shown in the paper) indicated that there were no differences by previous urban area of residence, only by current urban area of residence (which we therefore considered in our stratified analyses).

It is also likely that the association of maternal adult internal migration and child growth and nutritional health would have differed by *previous* geographic region, which was not available in the DHS. For example, children of rural-urban mothers who had migrated from coastal regions may have differed from those who had migrated from highland regions. The inclusion of ethnicity and maternal height as confounders should have allowed to adjust for this to some extent, as we found that *current* geographic region was associated with both, but there may have remained uncaptured differences. Additional analyses were carried out exploring associations of maternal adult internal migration and child outcomes by current region and patterns remained similar after adjusting for confounders (not shown in the paper).

The cross-sectional design of the DHS and the reliance of self-reported data means that the relationship between maternal migration and child nutritional outcomes cannot be demonstrated to be causal. Longitudinal data such as the Young Lives study would have allowed to explore causality and patterns as children age; however, the small sample and limited data did not allow us to address our research aims. Unmeasured confounding in the regression analyses may also have led to false conclusions both regarding the association between migration and child nutritional outcomes.

There may additionally be some ‘selection’ or ‘healthy migrant’ effects, whereby we see better-off or healthier individuals in the baseline population migrating. This may explain why we see better growth and nutritional health in migrants in some of our results. There is however limited evidence of these effects with internal migration; with most research to date having focussed on international migration. While mothers who have migrated from rural to urban areas do not appear taller than their non-migrant counterparts, which would have suggested better early socio-economic circumstances and/or health, they are more educated and from different ethnic backgrounds which could suggest greater wealth and/or greater potential for adaptation to the new setting. If there are indeed unobserved differences such as these which could not be accounted for then our findings may have overestimated the true effect of migration on health. This may nonetheless depend on the context of migration; it is possible that for mothers who migrate in situations of emergency (e.g conflict, climate, natural disaster) we would see a larger impact on child health compared to those who migrate for marital or other socio-economic reasons. It is likely that a lot of the women in our sample migrated for reasons related to marriage and this may have led to better socio-economic conditions and a change in environment which we were not able to capture with DHS data.

### Conclusion

While Peru has made great progress in improving child growth and reducing undernutrition overall, this varies by maternal and child internal migration history. Similarly to studies in other settings, we show that migrating may create unique nutritional profiles combining the risks and benefits of both rural and urban life and that both migration and rural/urban exposures are important in shaping child physical development and health in Peru. Being the child of a migrant in an urban area, regardless of whether urban or rural in origin, appears to be associated with lower weight growth and risk of overweight and even more so if the child was born before or during migration. In rural areas however, being the child of a migrant, particularly an urban-rural migrant was associated with better linear growth and reduced undernutrition. Nonetheless, even considering migration pathways, we still see large differences in growth and nutritional health between rural and urban children.

Although the prevalence of overweight, and inequalities therein, are relatively low in Peruvian children under 5 years, other research at older ages indicates these outcomes will likely increase, as will associated disease. It is unclear whether rural-urban migration may impact children’s physical development and physiology in other ways, and further investigations should explore other differences, for example in body composition and hormonal profiles. Further research into which factors mediate the association between maternal migration and child nutritional health would also help identify areas amenable to policies and interventions to improve child health in settings with high internal migration like Peru.

Interventions to improve child nutritional health could take into consideration maternal and child internal migration history, as well as different aspects of malnutrition which could exist within the same groups of children or the same households. As Peru continues to go through its nutritional and economic transition, we may see further declines in stunting, but a greater burden of obesity and metabolic disease emerge. Nonetheless, with recent developments related to the SARS-Cov-2 pandemic and as the climate crisis evolves, there could also be a widening of socio-economic inequalities and reversal of any progress made so far [19, 20, 78].

## Supplementary Information

**Additional file 1.**

## Data Availability

The datasets analysed during the current study are available in the USAID Demographic and Health Survey Program data repository at https://dhsprogram.com/Data/.

## Abbreviations

DHS: Demographic and Health Surveys
LMICs: low- and middle-income countries
ENDES: Encuesta Demográfica y de Salud Familiar
INEI: Instituto Nacional de Estadística e Informática
IRB: Institutional Review Board
HAZ: Height-for-age z scores
WAZ: Weight-for-age z scores
WHZ: Weight-for-height z scores
WHO: World Health Organization
SD: Standard Deviations
