# Supplementary material for: Maternal internal migration and child growth and nutritional health in Peru: an analysis of the demographic and health surveys from 1991 to 2017
Source: BMC Public Health. 2022 Jan 6;22:37. doi: 10.1186/s12889-021-12452-7 (PMC8734138; doi:10.1186/s12889-021-12452-7)
Supplement: Supplementary file 1 — Additional file 1. [file 12889_2021_12452_MOESM1_ESM.docx]

Additional File 1

**Maternal internal migration and child growth and nutritional health in Peru: an analysis of the Demographic and Health Surveys from 1991 to 2017.**

Rougeaux E, Miranda JJ, Fewtrell M, Wells JC.

| Table A1. Change in growth and nutritional outcomes in children under 5 years in the Peru DHS between 1991 to 2017 (marginal predictions and p-values from survey-weighted pooled data regression analyses) | | | | | | |
| --- | --- | --- | --- | --- | --- | --- |
| **I. Growth** | | | | | | |
|  | Height-for-age | | Weight-for-age | | Weight-for-height | |
|  | *Change 1991 to 2017*  *Z score (95 % CI)* | *Difference p-value* | *Change 1991 to 2017*  *Z score (95 % CI)* | *Difference p-value* | *Change 1991 to 2017*  *Z score (95 % CI)* | *Difference p-value* |
| Urban non-migrant | +0.8 (0.6; 0.9) | < 0.001 | +0.6 (0.5; 0.7) | < 0.001 | +0.2 (0.0; 0.3) | 0.005 |
| Urban-urban migrant | +0.5 (0.4; 0.6) | < 0.001 | +0.3 (0.2; 0.4) | < 0.001 | -0.1 (-0.2; 0.0) | 0.218 |
| Rural-urban migrant | +1.0 (0.8; 1.1) | < 0.001 | +0.5 (0.3; 0.6) | < 0.001 | -0.2 (-0.4; -0.1) | 0.001 |
| Rural non-migrant | +0.6 (0.4; 0.7) | < 0.001 | +0.3 (0.2; 0.4) | < 0.001 | -0.1 (-0.3; 0.0) | 0.030 |
| Rural-rural migrant | +0.5 (0.3; 0.7) | < 0.001 | +0.2 (0.1; 0.4) | 0.009 | -0.1 (-0.3; 0.0) | 0.131 |
| Urban-rural migrant | +0.7 (0.5; 0.9) | < 0.001 | +0.3 (0.1; 0.4) | 0.001 | -0.2 (-0.4; -0.1) | 0.001 |
| *Total* | +0.8 (0.7; 0.9) | < 0.001 | +0.5 (0.4; 0.6) | < 0.001 | 0.0 (-0.1; 0.1) | 0.869 |
| **II. Nutritional status** | | | | | | |
|  | Stunting | | Underweight | | Overweight | |
|  | *Change 1991 to 2017*  *Percent point (95 % CI)* | *Difference p-value* | *Change 1991 to 2017 Percent point (95 % CI)* | *Difference p-value* | *Change 1991 to 2017*  *Percent point (95 % CI)* | *Difference p-value* |
| Urban non-migrant | -22.5 (-26.0; -19.0) | < 0.001 | -4.9 (-6.7; -3.0) | < 0.001 | +2.5 (-0.1; 5.1) | 0.062 |
| Urban-urban migrant | -17.3 (-20.7; -13.8) | < 0.001 | -3.1 (-4.6; -1.7) | < 0.001 | -0.2 (-2.6; 2.3) | 0.882 |
| Rural-urban migrant | -34.4 (-40.2; -28.7) | < 0.001 | -4.9 (-7.8; -2.0) | 0.001 | -2.5 (-6.0; 1.1) | 0.172 |
| Rural non-migrant | -25.6 (-31.1; -20.2) | < 0.001 | -9.5 (-13.3; -5.8) | < 0.001 | -4.9 (-7.3; -2.6) | < 0.001 |
| Rural-rural migrant | -21.0 (-28.7; -13.4) | < 0.001 | -3.2 (-7.7; 1.3) | 0.158 | -1.3 (-4.9; 2.3) | 0.482 |
| Urban-rural migrant | -31.7 (-37.9; 25.5) | < 0.001 | -4.8 (-8.3; -1.4) | 0.006 | -2.7 (-5.7; 0.4) | 0.090 |
| *Total* | -27.5 (-30.0; -25.0) | < 0.001 | -6.4 (-7.8; -5.1) | < 0.001 | -0.2 (-1.5; 1.1) | 0.737 |

| Table A2. Regression of maternal adult internal migration on child growth, DHS 2017 (survey weighted, N = 10901; * = p-value <0.05) | | | | | | |
| --- | --- | --- | --- | --- | --- | --- |
| **I. Unadjusted results** | | | | | | |
|  | Height-for-age | | Weight-for-age | | Weight-for-height | |
| **Maternal migration status** | *Coefficient* | *95 % CI* | *Coefficient* | *95 % CI* | *Coefficient* | *95 % CI* |
| Urban non-migrant | Reference group | | | | | |
| Urban-urban migrant | -0.1 | -0.1; 0.0 | -0.1* | -0.2; -0.0 | -0.1* | -0.2; 0.0 |
| Rural-urban migrant | -0.3* | -0.4; -0.3 | -0.4* | -0.5; -0.3 | -0.2* | -0.3; -0.2 |
| Rural non-migrant | -1.0* | -1.1; -0.9 | -0.9* | -1.0; -0.8 | -0.5* | -0.6; -0.4 |
| Rural-rural migrant | -1.0* | -1.1; -0.9 | -0.9* | -1.0; -0.8 | -0.4* | -0.5; -0.3 |
| Urban-rural migrant | -0.6* | -0.7; -0.5 | -0.6* | -0.7; -0.5 | -0.4* | -0.5; -0.3 |
| **II. Adjusted for maternal schooling, ethnicity, height, age and child birth order, age, & sex** | | | | | | |
|  | Height-for-age | | Weight-for-age | | Weight-for-height | |
| **Maternal migration status** | *Coefficient* | *95 % CI* | *Coefficient* | *95 % CI* | *Coefficient* | *95 % CI* |
| Urban non-migrant | Reference group | | | | | |
| Urban-urban migrant | 0.0 | -0.1; 0.1 | -0.1* | -0.2; 0.0 | -0.1* | -0.2; 0.0 |
| Rural-urban migrant | -0.1 | -0.1; 0.0 | -0.2* | -0.3; -0.1 | -0.2* | -0.3; -0.1 |
| Rural non-migrant | -0.5* | -0.6; -0.4 | -0.6* | -0.6; -0.5 | -0.4* | -0.4; -0.3 |
| Rural-rural migrant | -0.5* | -0.5; -0.4 | -0.5* | -0.6; -0.4 | -0.3* | -0.4; -0.2 |
| Urban-rural migrant | -0.3* | -0.4; -0.2 | -0.4* | -0.5; -0.3 | -0.3* | -0.4; -0.2 |

| Table A3. Regression of maternal adult internal migration on child nutritional status, DHS 2017 (survey weighted, N = 10901; * = p-value < 0.05) | | | | | | |
| --- | --- | --- | --- | --- | --- | --- |
| **I. Unadjusted results** | | | | | | |
|  | Stunting | | Underweight | | Overweight | |
| **Maternal migration status** | *Odds ratio* | *95 % CI* | *Odds ratio* | *95 % CI* | *Odds ratio* | *95 % CI* |
| Urban non-migrant | Reference group | | | | | |
| Urban-urban migrant | 1.2 | 0.9; 1.5 | 1.0 | 0.6; 1.6 | 0.8* | 0.6; 1.0 |
| Rural-urban migrant | 1.8* | 1.4; 2.4 | 1.8* | 1.1; 3.0 | 0.5* | 0.4; 0.7 |
| Rural non-migrant | 7.2* | 5.6; 9.4 | 5.6* | 3.5; 9.1 | 0.3* | 0.2; 0.4 |
| Rural-rural migrant | 6.4* | 5.0; 8.4 | 5.2* | 3.2; 8.4 | 0.3* | 0.2; 0.5 |
| Urban-rural migrant | 3.2* | 2.4; 4.4 | 3.2* | 1.9; 5.4 | 0.3* | 0.2; 0.5 |
| **II. Adjusted for maternal schooling, ethnicity, height, age and child birth order, age, & sex** | | | | | | |
|  | Stunting | | Underweight | | Overweight | |
| **Maternal migration status** | *Odds ratio* | *95 % CI* | *Odds ratio* | *95%CI* | *Odds ratio* | *95 % CI* |
| Urban non-migrant | Reference group | | | | | |
| Urban-urban migrant | 1.0 | 0.8; 1.3 | 0.9 | 0.6; 1.5 | 0.8 | 0.6; 1.1 |
| Rural-urban migrant | 1.0 | 0.7; 1.3 | 1.1 | 0.7; 1.9 | 0.6* | 0.4; 0.8 |
| Rural non-migrant | 3.0* | 2.2; 3.9 | 2.6* | 1.5; 4.4 | 0.4* | 0.2; 0.6 |
| Rural-rural migrant | 2.4* | 1.8; 3.2 | 2.2* | 1.3; 3.8 | 0.5* | 0.3; 0.8 |
| Urban-rural migrant | 1.7* | 1.3; 2.3 | 1.8* | 1.0; 3.0 | 0.4* | 0.2; 0.6 |

| Table A4. Regression of maternal adult internal migration on child growth by timing of migration, DHS 2017 (survey weighted, restricted to children born after migration N = 5284; * = p-value < 0.05) | | | | | | | |
| --- | --- | --- | --- | --- | --- | --- | --- |
| **I. Unadjusted results** | | | | | | | |
|  |  | Height-for-age | | Weight-for-age | | Weight-for-height | |
| **Maternal migration status** | **Time since migration** | *Coefficient* | *95 % CI* | *Coefficient* | *95 % CI* | *Coefficient* | *95 % CI* |
| Urban-urban migrant | *≤5 years (vs >5years)* | -0.1* | -0.3; 0.0 | -0.1 | -0.3; 0.0 | -0.1 | -0.2; 0.1 |
| Rural-urban migrant | *≤5 years (vs >5years)* | 0.0 | -0.1; 0.2 | 0.1 | -0.1; 0.3 | 0.1 | -0.1; 0.3 |
| Rural-rural migrant | *≤5 years (vs >5years)* | 0.2 | 0.0; 0.3 | 0.1 | -0.1; 0.3 | 0.0 | -0.2; 0.2 |
| Urban-rural migrant | *≤5 years (vs >5years)* | 0.2 | 0.0; 0.4 | 0.3* | 0.1; 0.5 | 0.2* | 0.0; 0.4 |
| **II. Adjusted for maternal schooling, ethnicity, height, age and child birth order, age, & sex** | | | | | |  |  |
|  |  | Height-for-age | | Weight-for-age | | Weight-for-height | |
| **Maternal migration status** | **Child timing of birth** | *Coefficient* | *95 % CI* | *Coefficient* | *95 % CI* | *Coefficient* | *95 % CI* |
| Urban-urban migrant | *≤5 years (vs >5years)* | -0.1 | -0.3; 0.0 | -0.1 | -0.3; 0.0 | -0.1 | -0.3; 0.1 |
| Rural-urban migrant | *≤5 years (vs >5years)* | -0.1 | -0.2; 0.1 | 0.0 | -0.2; 0.2 | 0.0 | -0.1; 0.2 |
| Rural-rural migrant | *≤5 years (vs >5years)* | 0.0 | -0.2; 0.2 | 0.0 | -0.2; 0.2 | 0.0 | -0.2; 0.2 |
| Urban-rural migrant | *≤5 years (vs >5years)* | 0.1 | -0.1; 0.2 | 0.2 | 0.0; 0.4 | 0.2 | 0.0; 0.4 |

| Table A5. Regression of maternal adult internal migration on child nutritional status by timing of migration, DHS 2017 (survey weighted, restricted to children born after migration N = 5284;* = p-value < 0.05) | | | | | | | |
| --- | --- | --- | --- | --- | --- | --- | --- |
| **I. Unadjusted results** | | | | | | | |
|  |  | Stunting | | Underweight | | Overweight | |
| **Maternal migration status** | **Time since migration** | *Odds ratio* | *95 % CI* | *Odds ratio* | *95 % CI* | *Odds ratio* | *95 % CI* |
| Urban-urban migrant | *≤5 years (vs >5years)* | 1.1 | 0.7; 1.7 | 1.0 | 0.5; 1.9 | 0.9 | 0.6; 1.5 |
| Rural-urban migrant | *≤5 years (vs >5years)* | 1.1 | 0.7; 1.8 | 1.0 | 0.4; 2.4 | 0.9 | 0.5; 1.8 |
| Rural-rural migrant | *≤5 years (vs >5years)* | 0.7 | 0.5; 1.0 | 1.1 | 0.5; 2.3 | 2.0 | 0.9; 4.2 |
| Urban-rural migrant | *≤5 years (vs >5years)* | 0.6* | 0.3; 1.0 | 0.6 | 0.2; 1.5 | 2.1 | 0.8; 5.9 |
| **II. Adjusted for maternal schooling, ethnicity, height, age and child birth order, age, & sex** | | | | | |  |  |
|  |  | Stunting | | Underweight | | Overweight | |
| **Maternal migration status** | **Child timing of birth** | *Odds ratio* | *95 % CI* | *Odds ratio* | *95 % CI* | *Odds ratio* | *95 % CI* |
| Urban-urban migrant | *≤5 years (vs >5years)* | 1.1 | 0.6; 1.8 | 0.9 | 0.4; 1.7 | 0.9 | 0.5; 1.6 |
| Rural-urban migrant | *≤5 years (vs >5years)* | 1.5 | 0.8; 2.6 | 1.3 | 0.5; 3.3 | 0.8 | 0.3; 1.9 |
| Rural-rural migrant | *≤5 years (vs >5years)* | 0.8 | 0.5; 1.2 | 1.0 | 0.4; 2.5 | 1.6 | 0.6; 4.2 |
| Urban-rural migrant | *≤5 years (vs >5years)* | 0.5 | 0.2; 1.1 | 0.8 | 0.3; 2.3 | 2.5 | 0.8; 8.2 |

| Table A6. Regression of maternal migration on child growth by child timing of birth, DHS 2017 (survey weighted, restricted to children of recent migrants [≤ 5 years since arrival]; N = 3647; * = p-value < 0.05) | | | | | | | |
| --- | --- | --- | --- | --- | --- | --- | --- |
| **I. Unadjusted results** | | | | | | | |
|  |  | Height-for-age | | Weight-for-age | | Weight-for-height | |
| **Maternal migration status** | **Child timing of birth** | *Coefficient* | *95 % CI* | *Coefficient* | *95 % CI* | *Coefficient* | *95 % CI* |
| Urban-urban migrant | *After maternal migration (vs before/during)* | 0.1 | 0.0; 0.2 | 0.2* | 0.0; 0.3 | 0.1 | -0.1; 0.2 |
| Rural-urban migrant | *After maternal migration (vs before/during)* | 0.3* | 0.1; 0.4 | 0.3* | 0.1; 0.5 | 0.2* | 0.0; 0.4 |
| Rural-rural migrant | *After maternal migration (vs before/during)* | 0.2 | 0.0; 0.3 | 0.1 | -0.1; 0.3 | 0.0 | -0.2; 0.2 |
| Urban-rural migrant | *After maternal migration (vs before/during)* | 0.2 | -0.1; 0.4 | 0.2 | 0.0; 0.4 | 0.2 | -0.1; 0.4 |
| **II. Adjusted for maternal schooling, ethnicity, height, age and child birth order, age, & sex** | | | | | |  |  |
|  |  | Height-for-age | | Weight-for-age | | Weight-for-height | |
| **Maternal migration status** | **Child timing of birth** | *Coefficient* | *95 % CI* | *Coefficient* | *95 % CI* | *Coefficient* | *95 % CI* |
| Urban-urban migrant | *After maternal migration (vs before/during)* | 0.1 | 0.0; 0.2 | 0.1 | 0.0; 0.3 | 0.1 | -0.1; 0.2 |
| Rural-urban migrant | *After maternal migration (vs before/during)* | 0.1 | 0.0; 0.3 | 0.2* | 0.0; 0.4 | 0.2* | 0.0; 0.4 |
| Rural-rural migrant | *After maternal migration (vs before/during)* | 0.0 | -0.2; 0.2 | 0.0 | -0.2; 0.2 | 0.0 | -0.2; 0.2 |
| Urban-rural migrant | *After maternal migration (vs before/during)* | 0.0 | -0.2; 0.1 | 0.0 | -0.2; 0.2 | 0.1 | -0.1; 0.3 |

| Table A7. Regression of maternal adult internal migration on child nutritional status by child timing of birth, DHS 2017 (survey weighted, restricted to children of recent migrants [≤ 5 years since arrival]; N = 3647; * = p-value < 0.05) | | | | | | | |
| --- | --- | --- | --- | --- | --- | --- | --- |
| **I. Unadjusted results** | | | | | | | |
|  |  | Stunting | | Underweight | | Overweight | |
| **Maternal migration group** | **Child timing of birth** | *Odds ratio* | *95 % CI* | *Odds ratio* | *95 % CI* | *Odds ratio* | *95 % CI* |
| Urban-urban migrant | *After maternal migration (vs before/during)* | 0.8 | 0.5; 1.2 | 1.2 | 0.5; 2.8 | 1.7 | 1.0; 3.0 |
| Rural-urban migrant | *After maternal migration (vs before/during)* | 0.7 | 0.4; 1.1 | 1.3 | 0.4; 3.6 | 1.7 | 0.7; 4.1 |
| Rural-rural migrant | *After maternal migration (vs before/during)* | 0.7 | 0.4; 1.1 | 1.0 | 0.4; 2.4 | 1.5 | 0.6; 4.0 |
| Urban-rural migrant | *After maternal migration (vs before/during)* | 1.0 | 0.7; 1.6 | 0.8 | 0.3; 1.7 | 0.7 | 0.3; 1.9 |
| **II. Adjusted for maternal schooling, ethnicity, height, age and child birth order, age, & sex** | | | | | |  |  |
|  |  | Stunting | | Underweight | | Overweight | |
| **Maternal migration group** | **Child timing of birth** | *Odds ratio* | *95 % CI* | *Odds ratio* | *95 % CI* | *Odds ratio* | *95 % CI* |
| Urban-urban migrant | *After maternal migration (vs before/during)* | 0.8 | 0.5; 1.3 | 1.2 | 0.4; 3.3 | 2.0* | 1.1; 3.6 |
| Rural-urban migrant | *After maternal migration (vs before/during)* | 1.0 | 0.5; 1.7 | 1.3 | 0.4; 4.2 | 2.4 | 1.0; 5.9 |
| Rural-rural migrant | *After maternal migration (vs before/during)* | 0.9 | 0.5; 1.5 | 0.8 | 0.3; 2.2 | 1.2 | 0.4; 3.6 |
| Urban-rural migrant | *After maternal migration (vs before/during)* | 0.8 | 0.4; 1.4 | 0.5 | 0.2; 1.0 | 0.7 | 0.3; 1.9 |

| Table A8. Regression of maternal adult internal migration on child growth by current area of residence, DHS 2017 (survey weighted, restricted to urban residents N = 7403; * = p-value < 0.05) | | | | | | | |
| --- | --- | --- | --- | --- | --- | --- | --- |
| **I. Unadjusted results** | | | | | | | |
|  |  | Capital/large city | | Small city | | Town | |
| **Child growth** | **Maternal migration status** | *Coefficient* | *95 % CI* | *Coefficient* | *95 % CI* | *Coefficient* | *95 % CI* |
| Height-for-age | *Urban non-migrant* | Reference group | | | | | |
|  | *Urban-urban migrant* | 0.1 | 0.0; 0.2 | -0.1 | -0.2; 0.0 | 0.3* | 0.2; 0.4 |
|  | *Rural-urban migrant* | -0.1 | -0.3; 0.1 | -0.4* | -0.5; -0.2 | 0.1 | -0.1; 0.2 |
| Weight-for-age | *Urban non-migrant* | Reference group | | | | | |
|  | *Urban-urban migrant* | 0.1 | 0.0; 0.3 | -0.2* | -0.3; 0.0 | 0.2* | 0.1; 0.3 |
|  | *Rural-urban migrant* | -0.1 | -0.3; 0.1 | -0.3* | -0.4; -0.1 | 0.0 | -0.1; 0.2 |
| Weight-for-height | *Urban non-migrant* | Reference group | | | | | |
|  | *Urban-urban migrant* | 0.1 | -0.1; 0.2 | -0.1* | -0.2; 0.0 | 0.1 | 0.0; 0.2 |
|  | *Rural-urban migrant* | -0.1 | -0.3; 0.1 | -0.1 | -0.3; 0.0 | 0.0 | -0.1; 0.1 |
| **II. Adjusted for maternal schooling, ethnicity, height, age and child birth order, age, & sex** | | | | | |  |  |
|  |  | Capital/large city | | Small city | | Town | |
| **Child growth** | **Maternal migration status** | *Coefficient* | *95 % CI* | *Coefficient* | *95 % CI* | *Coefficient* | *95 % CI* |
| Height-for-age | *Urban non-migrant* | Reference group | | | | | |
|  | *Urban-urban migrant* | 0.1* | 0.0; 0.2 | -0.1 | -0.2; 0.0 | 0.2* | 0.1; 0.3 |
|  | *Rural-urban migrant* | 0.1 | -0.1; 0.3 | -0.1 | -0.3; 0.0 | 0.1* | 0.0; 0.3 |
| Weight-for-age | *Urban non-migrant* | Reference group | | | | | |
|  | *Urban-urban migrant* | 0.1 | 0.0; 0.3 | -0.2* | -0.3; -0.1 | 0.1 | 0.0; 0.2 |
|  | *Rural-urban migrant* | -0.1 | -0.3; 0.1 | -0.2* | -0.3; 0.0 | 0.1 | -0.1; 0.2 |
| Weight-for-height | *Urban non-migrant* | Reference group | | | | | |
|  | *Urban-urban migrant* | 0.1 | -0.1; 0.2 | -0.2* | -0.3; 0.0 | 0.0 | -0.1; 0.2 |
|  | *Rural-urban migrant* | -0.2 | -0.4; 0.1 | -0.1 | -0.3; 0.0 | 0.0 | -0.1; 0.1 |

| Table A9. Regression of maternal adult internal migration on child nutritional status by current area of residence, DHS 2017 (survey weighted, restricted to urban residents N = 7403; * = p-value < 0.05) | | | | | | | | |
| --- | --- | --- | --- | --- | --- | --- | --- | --- |
| **I. Unadjusted results** | | | | | | | | |
|  |  | Capital/large city | | Small city | | Town | |  |
| **Child nutritional status** | **Maternal migration status** | *Odds ratio* | *95 % CI* | *Odds ratio* | *95 % CI* | *Odds ratio* | *95 % CI* |  |
| Stunting | *Urban non-migrant* | Reference group | | | | | | |
|  | *Urban-urban migrant* | 0.9 | 0.5; 1.7 | 1.2 | 0.8; 1.8 | 0.5* | 0.4; 0.7 |  |
|  | *Rural-urban migrant* | 0.4 | 0.1; 1.7 | 1.9* | 1.2; 2.9 | 0.8 | 0.5; 1.1 |  |
| Underweight | *Urban non-migrant* | Reference group | | | | | | |
|  | *Urban-urban migrant* | 0.3 | 0.1; 1.6 | 0.9 | 0.4; 1.8 | 0.4* | 0.2; 0.8 |  |
|  | *Rural-urban migrant* | 0.3 | 0.0; 2.5 | 1.6 | 0.8; 3.3 | 0.6 | 0.3; 1.2 |  |
| Overweight | *Urban non-migrant* | Reference group | | | | | | |
|  | *Urban-urban migrant* | 1.0 | 0.7; 1.5 | 0.9 | 0.6; 1.4 | 1.0 | 0.7; 1.5 |  |
|  | *Rural-urban migrant* | 0.7 | 0.4; 1.2 | 0.8 | 0.5; 1.3 | 0.6 | 0.4; 1.0 |  |
| **II. Adjusted for maternal schooling, ethnicity, height, age and child birth order, age, & sex** | | | | | |  |  | |
|  |  | Capital/large city | | Small city | | Town | |  |
| **Child nutritional status** | **Maternal migration status** | *Odds ratio* | *95 % CI* | *Odds ratio* | *95 % CI* | *Odds ratio* | *95 % CI* |  |
| Stunting | *Urban non-migrant* | Reference group | | | | | | |
|  | *Urban-urban migrant* | 0.8 | 0.4; 1.5 | 1.2 | 0.7; 2.0 | 0.6* | 0.4; 0.8 |  |
|  | *Rural-urban migrant* | 0.2* | 0.0; 0.9 | 1.2 | 0.7; 2.1 | 0.6* | 0.4; 0.9 |  |
| Underweight | *Urban non-migrant* | Reference group | | | | | | |
|  | *Urban-urban migrant* | 0.4 | 0.0; 3.4 | 1.1 | 0.5; 2.3 | 0.5* | 0.3; 0.9 |  |
|  | *Rural-urban migrant* | 0.4 | 0.0; 4.0 | 1.4 | 0.6; 3.0 | 0.5 | 0.2; 1.1 |  |
| Overweight | *Urban non-migrant* | Reference group | | | | | | |
|  | *Urban-urban migrant* | 1.1 | 0.7; 1.6 | 0.8 | 0.5; 1.3 | 0.9 | 0.6; 1.3 |  |
|  | *Rural-urban migrant* | 0.6 | 0.3; 1.3 | 0.9 | 0.5; 1.5 | 0.7 | 0.4; 1.2 |  |

| Table A10. Regression of the mother and child double burden of malnutrition** and maternal adult internal migration, DHS 2017 (survey weighted, N = 10901; * = p-value < 0.05; ** = defined as mother overweight/obese and child stunted/underweight/wasted or child stunted and overweight) | | |
| --- | --- | --- |
| **I. Unadjusted results** | | |
|  | *Odds ratio* | *95 % CI* |
| Urban non-migrant | Reference group | |
| Urban-urban migrant | 1.2 | 0.9; 1.7 |
| Rural-urban migrant | 1.7* | 1.2; 2.3 |
| Rural non-migrant | 3.9* | 2.9; 5.3 |
| Rural-rural migrant | 4.4* | 3.3; 6.0 |
| Urban-rural migrant | 2.3* | 1.6; 3.3 |
| **II. Adjusted for maternal schooling, ethnicity, height, age and child birth order, age, & sex** | | |
|  | *Odds ratio* | *95 % CI* |
| Urban non-migrant | Reference group | |
| Urban-urban migrant | 1.0 | 0.7; 1.3 |
| Rural-urban migrant | 0.9 | 0.6; 1.2 |
| Rural non-migrant | 1.6* | 1.1; 2.3 |
| Rural-rural migrant | 1.6* | 1.1; 2.3 |
| Urban-rural migrant | 1.2 | 0.8; 1.8 |
